# Dataset of structure–activity relationships in Pd/ZrO_2_–TiO_2_ catalysts for furfural reductive amination: Batch vs Operando ATR-FTIR

**DOI:** 10.1016/j.dib.2026.113011

**Published:** 2026-06-25

**Authors:** Alex A. Fernández-Andrade, Katherine A. Arriagada-Fuentes, Juan Pablo Parra, Cristian H. Campos, Luis E. Arteaga-Pérez

**Affiliations:** a Laboratory of Thermal and Catalytic Processes (LPTC-UBB), Department of Process Engineering and Bioproducts, Engineering Faculty, Universidad del Bio-Bio, Concepción, 4030000, Chile; b Carbon and Catalysis Laboratory (CarboCat), Department of Chemical Engineering, Faculty of Engineering, Universidad de Concepcion, Chile; c Department of Chemical Engineering, Faculty of Engineering, Universidad de Concepcion, Concepcion, 4070409, Chile; d Universidad Andres Bello sede Concepción, Facultad de Ciencias Exactas, Departamento de Ciencias Químicas, Autopista Concepción-Talcahuano, 7100, Talcahuano, Chile

**Keywords:** Furfural amination, Furfurylamines, Bifunctional catalyst, Pd/ZrO_2_–TiO_2_

## Abstract

Furfural (FUR) is one of the main chemical platforms derived from lignocellulosic biomass and a relevant precursor of value-added compounds such as secondary amines. Among the available synthetic routes, the direct reductive amination of FUR with aniline (ANI) is an attractive and environmentally favourable alternative to displace fossil-based processes. Nevertheless, under hydrogen atmosphere and in the presence of metal-containing catalysts, this transformation is frequently accompanied by parallel pathways, including hydrogenations of the carbonyl group, aromatic rings, and the formation of undesired alcohol derivatives. Therefore, current efforts are focused on the rational design of bifunctional catalysts able to increase the selectivity to the secondary amines while keeping a stable performance. However, there is a lack of readily available and detailed experimental data allowing the elucidation of structure-activity relationships of catalytic systems involving bifunctional mechanisms and under reaction conditions (in operando). This article presents a dataset for the reductive amination of FUR with ANI over Pd/ZrO_2_–TiO_2_ catalysts using two reaction systems: (i) traditional batch reactors and (ii) a reactor for *in operando* measurements via Fourier Transform Infrared Spectroscopy coupled with Attenuated Total Reflectance measurements (FTIR-ATR). The dataset includes catalyst characterization data for several Pd/ZrO_2_-TiO_2_ systems in which the Zr/Ti ratio was varied to modulate surface properties (acid density, distribution of Lewis sites, etc.). The applied techniques include N_2_ physisorption, X-ray diffraction (XRD), X-ray photoelectron spectroscopy (XPS), H_2_ temperature-programmed reduction (H_2_-TPR), ammonia temperature-programmed desorption (NH_3_-TPD), pyridine adsorption infrared spectroscopy (IR-Pyr), transmission electron microscopy (TEM), and Scanning transmission electron microscopy (STEM) coupled with energy-dispersive X-ray spectroscopy (EDX). In addition, the dataset on catalytic activity includes different reaction conditions (solvent media, temperature, pressure, concentration of reactants, intermediates, and N-substrates). Furthermore, reaction profiles acquired by operando FTIR-ATR were integrated with product identification and quantification by gas chromatography-mass spectrometry (GC–MS) and flame ionization (GC-FID). Raw files, processed data, and calculation sheets are provided to facilitate reuse, independent analysis, kinetic treatment, comparative evaluation with related catalytic systems, and data-driven approaches for understanding furfural reductive amination.

Specifications Table

**Table:** 

Subject	Engineering & Materials science
Specific subject area	Heterogeneous catalysis for the synthesis of biomass-derived amines using bifunctional systems
Type of data	Table, Figure, Raw and Processed.
Data collection	Catalytic data were obtained from furfural reductive amination reactions performed in pressurized batch reactors (4 and 20 mL) under controlled temperature, H_2_ pressure, and stirring. Reaction products were identified by GC–MS (PerkinElmer Clarus 690/QS8) and quantified by GC-FID (SRI 8610). Time-resolved data to assess the effect of acid sites were collected *in operando* via FTIR-ATR in a Thermo Scientific Nicolet iS20 spectrometer with DTGS detector integrated with a Top Plate Golden Gate Specac ATR cell with a diamond crystal. FTIR spectra were post-processed by baseline correction, multiplicative scattering correction (MSC), and normalization using the solvent peak area at 1022 cm^-1^. Data processing and reactor control was carried out through Omnic^™TM^ Specta v 9.16.
Data source location	• Institutions: University of Bío-Bío and University of Concepción • City/Town/Region: Concepción/Concepción/Región del Biobío • Country: Chile.
Data accessibility	Repository name: Dataset of furfural reductive amination with aniline on bifunctional Pd/ZrO_2_-TiO_2_ catalysts: Materials Characterization and Performance Data identification number: 10.17632/np54m5jkxd.1 Direct URL to data: https://data.mendeley.com/datasets/np54m5jkxd/1
Related research article	A. A. Fernández-Andrade, J.A. Vergara, D. Gómez, D. González-Vera, C. H. Campos, J. M. Rodríguez-Díaz, L. E. Arteaga-Pérez. Exploring the Bifunctional Role of Pd/ZrO_2_-TiO_2_ in the Production of Secondary Amines via Reductive Amination of Furfural. Applied Catalysis A: General 2025, 705, 120455.

## 1 Value of the Data

The dataset incorporates the characterization of several Pd/ZrO_2_–TiO_2_ catalysts with modulated acid properties via the Zr/Ti ratio, along with catalytic activity results on the performance of these systems during the reductive amination of furfural with aniline under batch and *in operando* conditions. Therefore, the data are highly valuable for developing structural descriptors associated with surface properties, active site identification, solvent roles, hysteric hindrance, and for optimizing materials synthesis.

In a broader context, the data are relevant for researchers in heterogeneous catalysis, biomass valorisation, and green chemistry because they enable detailed analysis of structure–activity relationships and support mechanistic interpretation of the role of Pd sites and surface acidity during the reaction. The combination of processed results and raw data deposited in Mendeley Data allows independent validation, comparison with related reductive amination systems, and the formulation of kinetic and mechanistic models.

Furthermore, the dataset constitutes a useful reference for applied and industrial research focused on sustainable process development, as it offers a reliable basis for the rational design of catalysts and future scale-up strategies aimed at producing value-added secondary amines from biomass-derived platforms.

## 2 Background

Furfural is recognized for its flexibility as a synthetic platform for fuels, fine chemicals, additives, pharmaceuticals, and more [[1], [2], [3]]. In this framework, the reductive amination of furfural over heterogeneous bifunctional catalysts is a promising and environmentally friendly route for the production of secondary N-furfurylamines [[4], [5], [6], [7], [8]]. Despite its potential, the process still faces important limitations related to the non-selective hydrogenation of the carbonyl group and, in some cases, the aromatic ring, which hinders product selectivity [9,10]. In this context, understanding how active sites behave under different reaction conditions is essential, since the balance between metallic and acid sites strongly influences catalytic performance during furfural amination [1,11,12]. Accordingly, this article provides an experimental dataset for several Pd/ZrO_2_–TiO_2_ catalytic systems with tailored acid properties and proven bifunctionality during the amination of furfural with aniline. The catalysts were fully characterized by several complementary techniques and evaluated under different solvent media, temperatures, pressures, reactant concentrations and amine substrates. These results provide a complete and systematic base of information that facilitates the understanding of furfural amination at a mechanistic level and support relevant information for further rational design of new catalytic systems.

The main purpose of publishing these data separately, without extensive interpretation, is to provide readers with the opportunity to perform their own analyses and derive independent conclusions regarding the role of active sites and operating conditions in furfural reductive amination. In addition, the dataset may be valuable for comparative studies, data-driven analysis, and future kinetic or mechanistic modelling (Table 6).

## 3 Data Description

### 3.1 Catalyst characterization

To facilitate access to the information, the data included in this section are published in an open access Mendeley Data repository, which includes data files in .XLS and .SPA formats and images as .TIFF files [13]. Here, readers will find folder and files summarizing the characterization results for the catalysts (Pd/X%ZrO_2_-TiO_2_). The data is grouped into several sub-folders including surface properties such as specific surface area (S_BET_), average pore volumes, pore sizes distribution measured by N_2_ physisorption at 77 K. In addition, reducibility analysis of surface metal species via temperature programmed reduction of H_2_ (TPR-H_2_) are provided along with the identification and quantification of acid sites. The information associated to surface acid titration include the profiles of ammonia temperature programmed desorption (TPD-NH_3_), mass spectra of N_2_, NH_3_, and H_2_O and infrared pyridine adsorption patterns recorded at different temperatures (IR-Pyr). Finally, data on catalyst structure and morphology, obtained from X-ray diffraction (XRD), transmission electron microscopy (TEM) images with associated spreadsheets for particle size distribution and histograms, scanning transmission electron microscopy (STEM) coupled with energy-dispersive X-ray spectroscopy (EDX), and X-ray photoelectron spectroscopy (XPS), are provided. Table 1 summarizes the results of the different characterization techniques for each catalyst utilized. The results of the characterization of reference catalyst (Pd/SiO_2_), as well as additional information on the Pd/ZrO_2_-TiO_2_ catalysts, are available in our prior work and in the Mendeley Data repository [9,13].

**Table 1:** Summary of the physicochemical properties of the catalysts. S_BET_ is the specific surface area, V_p_ is pore volume and BA is bulk acidity.

*Properties*	*Pd* /X%ZrO_2_-TiO_2_*Catalysts (X %wt. = 0.5 – 25)*					
	*0.5*	*3*	*7*	*10*	*15*	*25*
*S_BET_ (m^2^/g)*	*35*	*39*	*41*	*44*	*46*	*56*
*V_p_ (cm^3^ g^-1^)*	*0.11*	*0.12*	*0.12*	*0.12*	*0.13*	*0.12*
*Pore diameter (nm)*	*12.8*	*12.5*	*11.5*	*11.2*	*10.9*	*8.7*
*BA (µmol* NH_3_*g^-1^)*	*181.5±7.1*	*121.7±5.2*	*150.9±8.3*	*187.3±4.2*	*213.7±7.2*	*204.7±3.3*
*Weak acid Sites (µmol* NH_3_*g^-1^)*	*150.6*	*92.1*	*132.0*	*135.8*	*180.1*	*172.0*
*Medium acid sites (µmol* NH_3_*g^-1^)*	*30.9*	*29.7*	*18.8*	*51.5*	*33.6*	*32.6*
*Pd particle size (nm)*	*5.7±2.3*	*4.9±1.9*	*5.4±2.3*	*6.6 ± 3.0*	*5.0 ± 1.7*	*6.2 ± 1.6*
*Pd content (wt.%)*	*1.35±0.15*	*1.39±0.1*	*1.33±0.12*	*1.40±0.1*	*1.42±0.14*	*1.35±0.12*

### 3.2 Control reaction data

Table 2 presents the results of the control reactions for the reductive amination of furfural with aniline in the liquid phase and the hydrogenation of furfural over Pd/SiO_2_ and Pd/3%ZrO_2_-TiO_2_ catalysts. In addition, to facilitate the understanding of the results, a reaction scheme with the main products identified is shown. This scheme was constructed based on our findings and has two main steps: i) formation of the imine by condensation of FUR with ANI; and ii) hydrogenation of the imine (IME) to secondary amine (FFA).

**Table 2:** Exploratory furfural amination and hydrogenation reactions. The initial conditions were: C°_FUR_ = C^0^_ANI_ = 0.5 mol L^-1^; H_2_ pressure = 3 bar; temperature = 100 °C; time = 3 h; m_cat_ = 50 mg; V_R_= 3 mL.


*Exp.*	*Catalysts*	*X _FUR_ (%)*	*Y _IME_ (%)*	*Y _FFA_ (%)*	*Y _TFFA_ (%)*	*Y _FOL_ (%)*
*1*	*Non-Catalytic*	*65.5*	*63.5*	*0*	*0*	*0*
*2*	*3%ZrO_2_-TiO_2_*	*85.7*	*84.3*	*0*	*0*	*0*
*3*	*Pd/3%ZrO_2_-TiO_2_*	*99.6*	*65.7*	*19.7*	*13.9*	*0*
*4*	*Pd/SiO_2_*	*90.5*	*88.6*	*1.3*	*0.54*	*0.04*
*5*	*Pd/3%ZrO_2_-TiO_2_	*35.4*	*0*	*0*	*0*	*34.2*
*6*	*Pd/SiO_2_	*19.6*	*0*	*0*	*0*	*19.6*

*Hydrogenation reactions, X: conversion and Y: Yield.

The comparison between the support and Pd-containing catalysts shows that Pd promotes the hydrogenation steps of the reaction by participating in the dissociation of H_2_ [14]. While 3%ZrO_2_–TiO_2_ mainly led to IME formation, Pd/3%ZrO_2_–TiO_2_ produced FFA and TFFA. In addition, furfural hydrogenation tests confirmed the ability of Pd catalysts to reduce FUR to FOL under H_2_.

### 3.3 Effect of reaction conditions data

Table 3 provides the FUR conversion data for the reductive amination with ANI carried out in a batch system at different initial conditions of concentration, H_2_ pressure, and temperature.

**Table 3:** Screening reaction conditions for the reductive amination of FUR with ANI on Pd/ZrO_2_-TiO_2_. The reactions were performed with 0.5 mol L^-1^ FUR, 50 mg catalyst and Ter-amyl alcohol (TAA) for 120 min. Reaction volume = 3 mL.

*Conversion of furfural (%)*				
$P_{H_{2}}\left(bar\right)$	$C_{ANI}^{0}$*mol l^-1^*	Temperature ( °C)		
		*50*	*75*	*100*
***0.5***	***0.125***	*22.8*	*28.5*	*32.6*
	***0.25***	*46.6*	*49.6*	*50.8*
	***0.5***	*80.1*	*86.2*	*92.0*
***1***	***0.125***	*27.9*	*28.3*	*31.5*
	***0.25***	*46.7*	*53.7*	*53.1*
	***0.5***	*92.1*	*92.3*	*92.4*
***2***	***0.125***	*31.8*	*29.5*	*32.6*
	***0.25***	*48.5*	*42.9*	*52.4*
	***0.5***	*92.7*	*93.2*	*93.8*

Table 4, Table 5, Table 6, Table 7, Table 8 present the kinetic data of the amination reactions at different initial conditions of FUR concentration, ANI concentration, hydrogen pressure, and temperature. To ensure proper sampling at different times, these experiments were scaled-up to 20 mL reactor, and the amount of substrate was adjusted to maintain a constant substrate/catalyst ratio. The raw data and calculations are available in a Mendeley repository [13].

**Table 4:** Yield and selectivity of the reductive amination products of FUR with ANI on Pd/ZrO_2_-TiO_2_ under different reaction conditions (time = 120 min; m_cat_ = 125 mg; V_R_= 20 mL; Solvent = TAA).

*Parameter*	*Yield (Y, %)*			*Selectivity (S, %)*		
	$Y_{IME}$	$Y_{FFA}$	$Y_{TFFA}$	$S_{IME}$	$S_{FFA}$	$S_{TFFA}$
***FUR concentration (mol*l*^-1^)***						
*0.5*	*19.7*	*1.4*	*0.1*	*93.0*	*6.5*	*0.6*
*0.25*	*31.9*	*5.2*	*0.6*	*84.7*	*13.7*	*1.6*
*0.125*	*43.9*	*14.3*	*3.3*	*71.3*	*23.3*	*5.4*
***ANI concentration (mol*l*^-1^)***						
*0.125*	*19.7*	*1.4*	*0.1*	*93.0*	*6.5*	*0.6*
*0.25*	*32.5*	*2.2*	*0.5*	*92.3*	*6.4*	*1.4*
*0.5*	*41.1*	*2.7*	*2.9*	*88.0*	*5.7*	*6.3*
***H_2_ pressure (bar)***						
*2*	*13.0*	*2.2*	*0.2*	*84.4*	*14.4*	*1.2*
*1*	*19.7*	*1.4*	*0.1*	*93.0*	*6.5*	*0.6*
*0.5*	*16.4*	*2.1*	*0.1*	*87.9*	*11.5*	*0.7*
Temperature ( °C)						
*50*	*19.7*	*1.4*	*0.1*	*93.0*	*6.5*	*0.6*
*75*	*16.7*	*4.3*	*0.3*	*78.3*	*20.2*	*1.5*
*100*	*14.9*	*5.5*	*0.8*	*70.4*	*26.0*	*3.7*

**Table 5:** Temporal profiles of FUR conversion (X) and FFA yield (Y) using different initial concentrations of FUR on Pd/ZrO_2_-TiO_2_. The reactions were carried out at: C^0^_ANI_ = 0.125 mol L^-1^; H_2_ pressure = 1 bar; temperature = 50 °C; time = 120 min; m_cat_ = 125 mg; V_R_= 20 mL.

*Effect of initial concentration of furfural*			
*Time (*min*)*	$X_{FUR}\;\left(\%\right)$		
	$C_{FUR}^{0}$*=0.5mol l^-1^*	$C_{FUR}^{0}$*=0.25mol l^-1^*	$C_{FUR}^{0}$*=0.125mol l^-1^*
*0*	*0*	*0*	*0*
*2*	*23*	*36*	*88*
*6*	*22*	*52*	*87*
*10*	*23*	*59*	*86*
*20*	*22*	*58*	*87*
*30*	*23*	*59*	*86*
*60*	*22*	*57*	*88*
*120*	*24*	*58*	*89*

***Time (*min*)***	$Y_{\mathrm{FFA}\;}(\%)$		

$C_{\mathrm{FUR}}^{0}$***=0.5mol*l*^-1^***	$C_{\mathrm{FUR}}^{0}$***=0.25mol*l*^-1^***	$C_{\mathrm{FUR}}^{0}$***=0.125mol*l*^-1^***

*0*	*0*	*0*	*0*
*2*	*0.2*	*1.0*	*0.7*
*6*	*0.4*	*0.6*	*0.9*
*10*	*0.5*	*1.1*	*1.3*
*20*	*0.5*	*1.6*	*2.4*
*30*	*0.7*	*1.8*	*3.7*
*60*	*0.9*	*2.7*	*9.0*
*120*	*1.4*	*5.2*	*14.3*

**Table 6:** Temporal profiles of FUR conversion (X) and FFA yield (Y) using different initial concentrations of ANI on Pd/ZrO_2_-TiO_2_. The reactions were carried out at: C°_FUR_ = 0.5 mol L^-1^; H_2_ pressure = 1 bar; temperature = 50 °C; time = 120 min; m_cat_ = 125 mg; V_R_= 20 mL.

*Effect of initial concentration of Aniline*			
*Time (*min*)*	$X_{FUR}\;\left(\%\right)$		
	$C_{ANI}^{0}$*=0.125mol l^-1^*	$C_{ANI}^{0}$*=0.25mol l^-1^*	$C_{ANI}^{0}$*=0.5mol l^-1^*
*0*	*0*	*0*	*0*
*2*	*23*	*52*	*91*
*6*	*22*	*55*	*89*
*10*	*23*	*52*	*86*
*20*	*22*	*57*	*88*
*30*	*23*	*58*	*92*
*60*	*22*	*63*	*91*
*120*	*24*	*51*	*88*

***Time (*min*)***	$Y_{\mathrm{FFA}}\;\left(\%\right)$		

$C_{\mathrm{ANI}}^{0}$***=0.125mol*l*^-1^***	$C_{\mathrm{ANI}}^{0}$***=0.25mol*l*^-1^***	$C_{\mathrm{ANI}}^{0}$***=0.5mol*l*^-1^***

*0*	*0*	*0*	*0*
*2*	*0.2*	*0.2*	*0.1*
*6*	*0.4*	*0.3*	*0.2*
*10*	*0.5*	*0.5*	*0.5*
*20*	*0.5*	*0.6*	*0.7*
*30*	*0.7*	*0.8*	*1.9*
*60*	*0.9*	*1.2*	*2.4*
*120*	*1.4*	*2.2*	*2.7*

**Table 7:** Temporal profiles of FUR conversion (X) and FFA yield (Y) using different hydrogen pressures on Pd/ZrO_2_-TiO_2_. The reactions were carried out at: C°_FUR_ = 0.5 mol L^-1^; C^0^_ANI_ = 0.125 mol L^-1^; temperature = 50 °C; time = 120 min; m_cat_ = 125 mg; V_R_= 20 mL.

*Effect of hydrogen pressure*			
*Time (*min*)*	$X_{FUR\;}\left(\%\right)$		
	$p_{H_{2}}^{0}$*=2.0 bar*	$p_{H_{2}}^{0}$*=1.0 bar*	$p_{H_{2}}^{0}$*=0.5 bar*
*0*	*0*	*0*	*0*
*2*	*37*	*23*	*30*
*6*	*42*	*22*	*33*
*10*	*40*	*23*	*35*
*20*	*41*	*22*	*35*
*30*	*42*	*23*	*23*
*60*	*41*	*22*	*36*
*120*	*41*	*24*	*35*

***Time (*min*)***	$Y_{\mathrm{FFA}}\;\left(\%\right)$		

$p_{H_{2}}^{0}$***=2.0 bar***	$p_{H_{2}}^{0}$***=1.0 bar***	$p_{H_{2}}^{0}$***=0.5 bar***

*0*	*0*	*0*	*0*
*2*	*0.1*	*0.2*	*0.2*
*6*	*0.1*	*0.4*	*0.2*
*10*	*0.3*	*0.5*	*0.2*
*20*	*0.4*	*0.5*	*0.4*
*30*	*0.7*	*0.6*	*0.5*
*60*	*1.0*	*0.9*	*0.9*
*120*	*2.2*	*1.4*	*2.1*

**Table 8:** Temporal profiles of FUR conversion (X) and FFA yield (Y) using different temperatures on Pd/ZrO_2_-TiO_2_. The reactions were carried out at: C°_FUR_ = 0.5 mol L^-1^; C^0^_ANI_ = 0.125 mol L^-1^; H_2_ pressure = 1 bar; time = 120 min; m_cat_ = 125 mg; V_R_= 20 mL.

*Effect of reaction temperature*			
*Time (*min*)*	$X_{FUR}\left(\%\right)$		
	$T=50{\;}^{\circ }C$	$T=75{\;}^{\circ }C$	$T=100{\;}^{\circ }C$
*0*	*0*	*0*	*0*
*2*	*23*	*24*	*23*
*6*	*22*	*32*	*25*
*10*	*23*	*31*	*31*
*20*	*22*	*30*	*31*
*30*	*23*	*31*	*30*
*60*	*22*	*32*	*30*
*120*	*24*	*31*	*30*

***Time (*min*)***	$Y_{\mathrm{FFA}}\;\left(\%\right)$		

$T=50{\;}^{\circ }C$	$T=75{\;}^{\circ }C$	$T=100{\;}^{\circ }C$

*0*	*0*	*0*	*0*
*2*	*0.2*	*0.2*	*0.4*
*6*	*0.4*	*0.2*	*0.5*
*10*	*0.5*	*0.2*	*0.6*
*20*	*0.5*	*0.4*	*1.6*
*30*	*0.7*	*0.9*	*1.9*
*60*	*0.9*	*2.2*	*4.0*
*120*	*1.4*	*4.3*	*5.5*

The data presented in Table 4, Table 5, Table 6, Table 7, Table 8 allow for a descriptive and general interpretation of the effect of reaction conditions on FFA formation. FFA production increased at lower initial FUR concentrations, likely due to lower saturation of the active sites. Conversely, a higher concentration of ANI favoured FFA formation, possibly by shifting the condensation equilibrium toward greater IME production. Likewise, increased H2 pressure and temperature promoted FFA formation, which may be associated with greater availability of activated hydrogen and an acceleration of the hydrogenation steps.

Since the reaction medium and the amine-donating compound can influence the reductive amination of furfural, the performance of solvents of different natures was studied in combination with various N-substrates as a nitrogen source, and the results are presented in Table 9.

**Table 9:** FUR conversion (X) and FFA yield (Y) using different solvents (protic and aprotic) and N-Substrates on Pd/ZrO_2_-TiO_2_. The reactions were carried out at: C°_FUR_ = 0.5 mol L^-1^; C°_N-Substrate_ = 0.5 mol L^-1^; H_2_ pressure = 1 bar; time = 120 min; m_cat_ = 125 mg; V_R_= 20 mL; Methanol as solvent.

Solvent	X_FUR_ (%)	Y_FFA_ (%)	N-Substrate	X_FUR_ (%)	Y_FFA_ (%)
Ethanol	46.1	18.8	4‑chloro-3-(trifluoromethyl)aniline	79.3	33.0
Methanol	92.6	31.5	4-chloroaniline	87.7	53.8
Tert-amyl alcohol	80.2	3.5	p-anisidine	95.8	16.2
Toluene	87.6	26.1	3-fluoroaniline	92.6	76.4
Dodecane	53.5	10.6	4-(trifluoromethyl)aniline	88.5	34.1
Iso-propyl alcohol	91.6	31.7	4-aminobenzonitrile	72.1	18.9
			p-toluidine	96.9	34.6
			2-chloroaniline	94.2	25.5

### 3.4 Effect of acid sites using ATR-FTIR operando

Based on our previously published study on the effect of solvents on the reductive amination of FUR [15], methanol was chosen for this stage of the investigation. Table 10 contains data on the amination of FUR with ANI using Pd catalysts supported on ZrO_2_-TiO_2_ with different Zr/Ti ratios.

**Table 10:** Results of reductive amination of FUR with ANI over catalysts with different Zr loading. Reaction conditions were: C^0^_ANI_ = C° _FUR_ = 0.5 mol L^-1^; T = 100 °C; P_H2_ =3 bar; t_R_ =120 min; V_R_ = 10 mL and methanol.

Catalyst	Y_IME_ (%)	Y_FFA_ (%)	Y_TFFA_ (%)	Y_FOL_ (%)	S_IME_ (%)	S_FFA_ (%)	S_TFFA_ (%)	S_FOL_ (%)	TOF (S^-1^)
Pd/0.5%ZrO_2_-TiO_2_	24.5	34.2	4.2	5.1	36.5	50.3	5.7	7.5	5.55
Pd/3%ZrO_2_-TiO_2_	38.6	22.4	10.9	8.9	49.2	27.7	12.1	11.0	0.78
Pd/7%ZrO_2_-TiO_2_	37.9	20.7	13.2	10.4	47.7	25.2	14.4	12.7	0.98
Pd/10%ZrO_2_-TiO_2_	29.7	30.6	8.5	6.9	40.7	40.4	9.8	9.1	5.16
Pd/15%ZrO_2_-TiO_2_	28.3	51.6	5.3	4.8	31.0	57.3	6.4	5.3	6.68
Pd/25%ZrO_2_-TiO_2_	24.3	59.1	7.8	6.5	23.9	60.5	8.9	6.7	3.08

The reported values correspond to the average of the measurements, with relative deviations of between 3.1% and 4.5% for yields, between 2.5% and 4.0% for selectivities, and between 1.0 and 1.5 S^-1^ for TOF values. Furthermore, to make the above calculations even transparent, the data on the evolution of the compounds (normalized area over time) used to construct the reaction profiles are presented in Tables 11 through 14. Complete information on the reaction profiles can be found in the Mendeley Data repository [13].

**Table 11:** Normalized peak area of FUR as a function of time. Reaction conditions were: C^0^_ANI_ = C° _FUR_ = 0.5 mol L^-1^; T = 100 °C; P_H2_ =3 bar; t_R_ =120 min; V_R_ = 10 mL and methanol.

*Time (*min*)*	*Normalized area of FUR (a.u.)* *Reference peak: 1675cm^-1^ (C=O)*					
	*Pd* /X%ZrO_2_-TiO_2_*Catalysts (X% = 0.5 – 25)*					
	*0.5*	*3*	*7*	*10*	*15*	*25*
*0*	*—–*	*—–*	*—–*	*—–*	*—–*	*—–*
*10*	*0.9169*	*0.8019*	*0.1523*	*0.2363*	*0.7051*	*0.5349*
*20*	*0.1212*	*0.5435*	*0.5266*	*0.6612*	*0.3016*	*0.3236*
*30*	*0.0373*	*0.3169*	*0.2597*	*0.2606*	*0.0351*	*0.1231*
*60*	*0.0311*	*0.2204*	*0.0160*	*0.0519*	*0.0397*	*0.0146*
*90*	*0.0352*	*0.2143*	*0.0175*	*0.0192*	*0.0113*	*0.0109*
*120*	*0.0280*	*0.1707*	*0.0176*	*0*	*0*	*0*

**Table 12:** Normalized area of ANI as a function of time. Reaction conditions were: C^0^_ANI_ = C° _FUR_ = 0.5 mol L^-1^; T = 100 °C; P_H2_ =3 bar; t_R_ =120 min; V_R_ = 10 mL and methanol.

*Time (*min*)*	*Normalized area of ANI (a.u.)* *Reference peak: 1605cm^-1^ (C–N/aryl coupling)*					
	*Pd* /X%ZrO_2_-TiO_2_*Catalysts (X% = 0.5 – 25)*					
	*0.5*	*3*	*7*	*10*	*15*	*25*
*0*	*—–*	*—–*	*—–*	*—–*	*—–*	*—–*
*10*	*0.7439*	*0.5837*	*0.0837*	*0.1759*	*0.4384*	*0.8525*
*20*	*0.9689*	*0.6076*	*0.6865*	*0.6734*	*0.6710*	*0.3478*
*30*	*0.6989*	*0.3732*	*0.2425*	*0.2299*	*0.1719*	*0.1863*
*60*	*0.0399*	*0.1388*	*0.0296*	*0.0616*	*0.1123*	*0.0534*
*90*	*0.0133*	*0.0739*	*0.0118*	*0.0593*	*0.0188*	*0.0568*
*120*	*0*	*0.0189*	*0.0086*	*0.0133*	*0*	*0.0677*

**Table 13:** Normalized area of IME as a function of time. Reaction conditions were: C^0^_ANI_ = C° _FUR_ = 0.5 mol L^-1^; T = 100 °C; P_H2_ =3 bar; t_R_ =120 min; V_R_ = 10 mL and methanol.

*Time (*min*)*	*Normalized area of IME (a.u.)* *Reference peak: 1650cm^-1^ (IME, C=N)*					
	*Pd* /X%ZrO_2_-TiO_2_*Catalysts (X% = 0.5 – 25)*					
	*0.5*	*3*	*7*	*10*	*15*	*25*
*0*	*—–*	*—–*	*—–*	*—–*	*—–*	*—–*
*10*	*0.5655*	*0.5979*	*0.4597*	*0.5066*	*0.5622*	*0.8261*
*20*	*0.7571*	*0.9984*	*0.9131*	*0.8696*	*0.7679*	*0.9424*
*30*	*0.9386*	*0.8067*	*0.8192*	*0.9311*	*0.9056*	*0.9994*
*60*	*0.6675*	*0.6768*	*0.6132*	*0.4628*	*0.4291*	*0.5698*
*90*	*0.5806*	*0.5976*	*0.5648*	*0.3919*	*0.2247*	*0.3985*
*120*	*0.5968*	*0.6036*	*0.5072*	*0.3549*	*0.1034*	*0.4035*

**Table 14:** Normalized area of FFA as a function of time. Reaction conditions were: C^0^_ANI_ = C° _FUR_ = 0.5 mol L^-1^; T = 100 °C; P_H2_ =3 bar; t_R_ =120 min; V_R_ = 10 mL and methanol.

*Time (*min*)*	*Normalized area of FFA (a.u.)* *Reference peak: 1317cm^-1^ (FFA, N—H)*					
	*Pd* /X%ZrO_2_-TiO_2_*Catalysts (X% = 0.5 – 25)*					
	*0.5*	*3*	*7*	*10*	*15*	*25*
*0*	*—–*	*—–*	*—–*	*—–*	*—–*	*—–*
*10*	*0.4047*	*0.3091*	*0.4221*	*0.0249*	*0.3848*	*0.1899*
*20*	*0.7098*	*0.6105*	*0.6765*	*0.5231*	*0.6510*	*0.3971*
*30*	*0.8570*	*0.8483*	*0.7083*	*0.6546*	*0.8013*	*0.7414*
*60*	*0.8638*	*0.8842*	*0.8765*	*0.7611*	*0.9305*	*0.7742*
*90*	*0.9122*	*0.8814*	*0.9524*	*0.9362*	*0.9359*	*0.8953*
*120*	*0.9098*	*0.9202*	*0.9816*	*0.9391*	*0.9738*	*0.9617*

## 4 Experimental Design, Materials and Methods

### 4.1 Catalysts synthesis

**Fig. 1:**
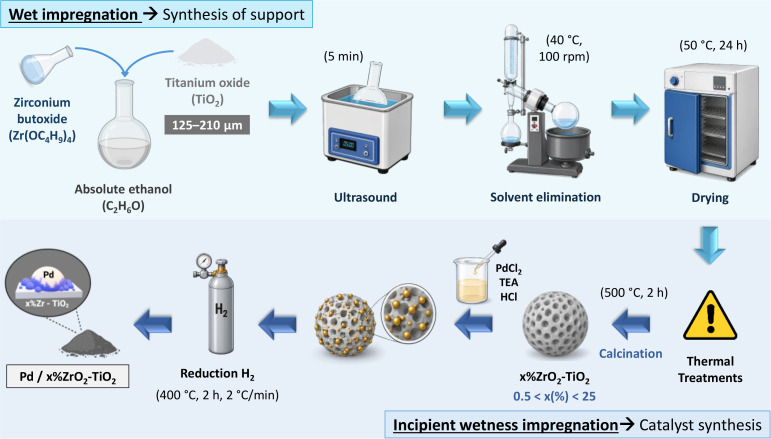
Schematic illustration of the two-step methodology used for the synthesis of Pd/x% ZrO_2_-TiO_2_ catalysts.

The catalysts were synthesized by wet impregnation of Pd onto ZrO_2_–TiO_2_ mixed-oxide supports with different ZrO_2_ contents, corresponding to 0.5, 3, 7, 10, 15, and 25 Zr wt% as shown in Fig. 1.

The support was prepared by wet impregnation of zirconium butoxide (Zr(OC_4_H_9_)_4_, CAS 1071–76–7, Sigma-Aldrich) onto titanium oxide (TiO_2_, CAS 13,463-67–7, Sigma-Aldrich), according to a previously reported procedure [9,15]. To do this, 2000 mL of absolute ethanol (C_2_H_6_O, CAS 64–17–5, Sigma-Aldrich) was added to a glass flask, along with the required amount of zirconium butoxide to achieve the desired ZrO_2_ loading. The suspension was initially homogenized in an ultrasonic bath for 5 min, after which TiO_2_ in the anatase phase, previously ground and sieved to a particle size between 125 and 210 µm, was added. Subsequently, the mixture was subjected to ultrasonication again for 5 min to promote adequate dispersion of the phases. The solvent was then removed in a rotary evaporator at 40 °C, maintaining continuous stirring at 100 rpm. The resulting solid was dried at 50 °C for 24 h and finally calcined in air at 500 °C for 2 h, using a flow rate of 50 mL min^-1^.

The Pd metal phase, with a nominal loading of 1.5% by weight, was incorporated via incipient wet impregnation using a PdCl_2_ precursor solution (Merck, CAS 7647–10–1, purity >99%) adjusted to pH 1.5, following the method described by Ortega et al. [16]. Finally, the impregnated solids were reduced in a stream of pure H_2_ (40 mL min^-1^, Air Liquide, 99.99%) at 400 °C for 2 h.

### 4.2 Catalyst characterization

Surface area, volume, and average pore size were estimated by N_2_ physisorption at 77 K in a Micromeritics Gemini VII 2390t by fitting the BET and BHJ models to the experimental data over a relative pressure range of 0.01 to 0.988. The reducibility of the catalyst component species was verified by temperature programmed reduction experiments (TPR-H_2_) up to 550 °C in a Micromeritics ASAP 2010 device under 50 ml min^-1^ of 5% v/v H_2_/Ar.

The bulk acidity and their strength were determined by the ammonia temperature-programmed desorption of ammonia (NH_3_-TPD) technique. The experiments were carried out by heating the sample from 100 to 750 °C with a ramp of 10 °C min^-1^, using the 3FLEX (Micromeritics) apparatus equipped with a TCD combined with a mass spectrometer (Pfeiffer Vacuum OmniStar GSD 320). The signals of NH_3_, H_2_O, N_2_ and N_2_O were recorded using an online mass spectrometer following specific fragments, *m/z*: 16, 18, 28 and 44, respectively. Desorption profiles and acid site density were normalized per gram of catalyst. The Infrared Pyridine adsorption study (IR-Pyr) was carried out by pretreating the sample disk at 400 °C for 12 h under vacuum conditions. The sample was then cooled to 150 °C and the reference spectrum was taken. Subsequently, the pyridine was brought into contact with the sample for 10 min. The measurements were carried out at three different temperatures (150, 250 and 350 °C) after 30 min at each temperature.

The composition of the crystalline phases was investigated by X-ray diffraction (XRD) on a Bruker D4 diffractometer with CuKα radiation (λ = 0.15418 nm) in a 2θ angular range from 3 to 90° The results were analyzed in the X'Pert HighScore Plus software by comparing the patterns with the Crystallography Open Database (COD). To determine the average size of metallic particles, the transmission electron microscopy (TEM) and high-resolution TEM (HR-TEM) techniques was employed with a JEOL JEM 1200 EXII electron microscope at 120 kV and a JEOL JEM 2010 electron microscope operating at 200 kV with a resolution point of 2.35 Å. The images were processed using the ImageJ software, to measure and count the Pd particles. The configuration of high-angle annular dark-field scanning transmission electron microscopy (STEM) and the energy-dispersive X-ray spectroscopy (EDX) were performed simultaneously for chemical analysis, using an Euro EA3000 (EuroVector) elemental analyser.

Finally, the chemical state of Pd was identified by X-ray photoelectron spectroscopy (XPS) in an instrument with a PHOIBOS 150 MCD 9 analyser from SPECS and a non-monochromatic Alkα X-ray energy of 1486.60 eV. The energy was corrected using the binding energy 103.4 eV of the Si 2p component for Pd/SiO_2_ and 459.0 eV for the Ti 2p of Pd/ZrO_2_-TiO_2_ catalysts. The spectra of the materials were collected after *ex situ* reduction at 400 °C for 120 min under H_2_.

### 4.3 Catalytic activity tests

**Fig. 2:**
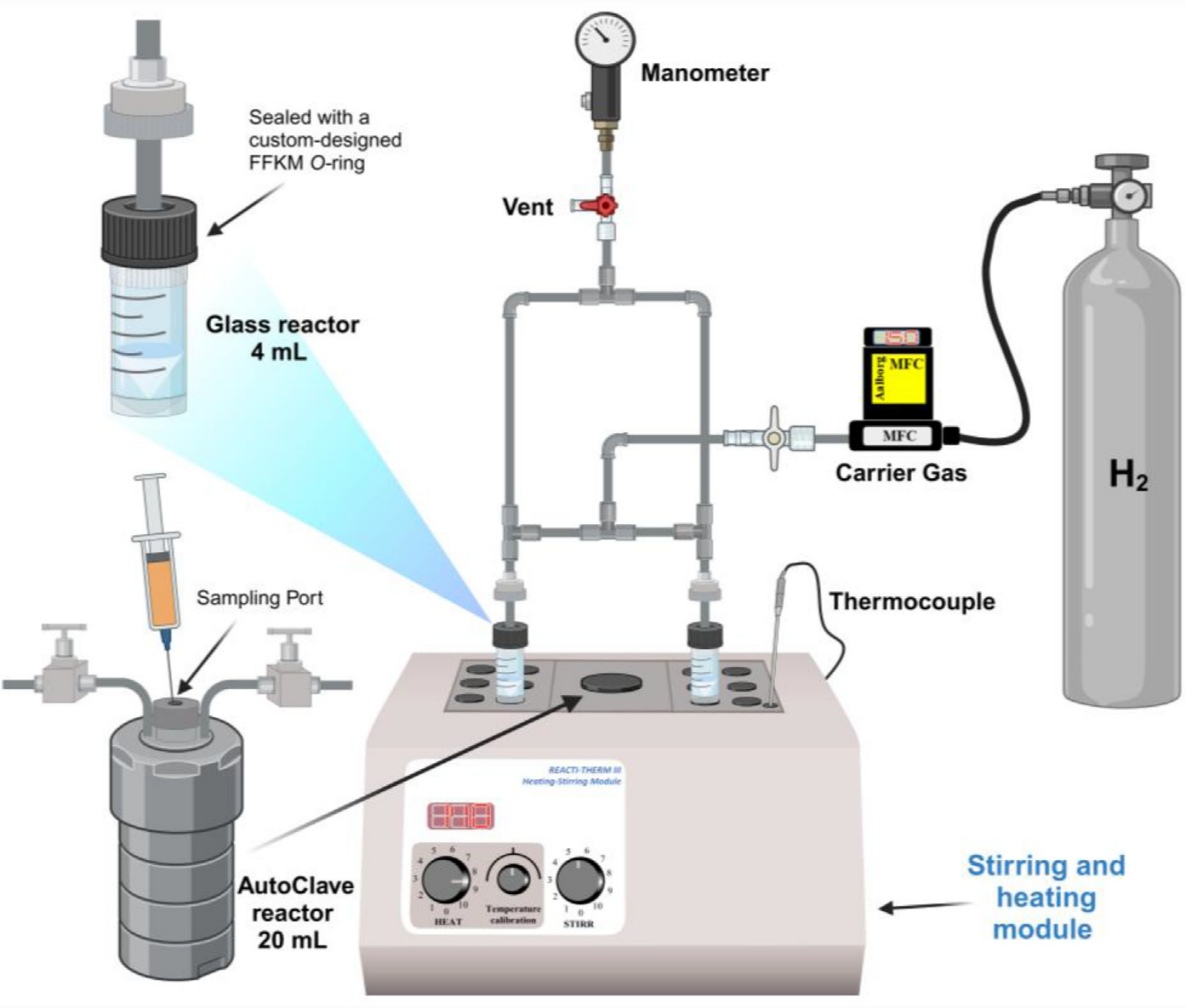
Reaction system used for control and kinetics measurements under batch and dynamic conditions with glass and autoclave reactors.

Control reactions of furfural (Sigma Aldrich®, CAS: 98–01–1), aniline (Sigma Aldrich®, CAS: 62–53–3), and H_2_ (Air Liquide, 99.99%) in tert‑amyl alcohol (Merck, CAS: 75–85–4) were carried out in a 4 mL autoclave type glass reactor placed in a Reacti-Therm™ (Thermofisher, USA) system with the configuration reported in Fig. 2 under the conditions reported in Table 15. Moreover, the same system and conditions were used for screening several solvent media: ethanol (Merck, CAS: 64–17–5), methanol (Merck, CAS: 67–56–1), tert‑amyl alcohol (Merck, CAS: 75–85–4), dodecane (Merck, CAS: 112–40–3), isopropyl alcohol (Merck, CAS: 67–63–0) and toluene (Merck, CAS: 108–88–3).

**Table 15:** Experimental conditions for control reactions with Pd/3%ZrO_2_-TiO_2_ and Pd/SiO_2_ catalysts. The tests were performed at 100 °C for 3 h in TAA as a solvent media and 50 mg of catalyst.

*Exp.*	*Catalysts*	*FUR (moll^-1^)*	*ANI (moll^-1^)*	*P H_2_ (bar)*	*V_R_ (mL)*
*1*	*Non-catalyst*	*0.5*	*0.5*	*1*	*3*
*2*	*3%ZrO_2_-TiO_2_*	*0.5*	*0.5*	*1*	*3*
*3*	*Pd/3%ZrO_2_-TiO_2_*	*0.5*	*0.5*	*1*	*3*
*4*	*Pd/SiO_2_*	*0.5*	*0.5*	*1*	*3*
*5*	*Pd/3%ZrO_2_-TiO_2_*	*0.5*	*0*	*1*	*3*
*6*	*Pd/SiO_2_*	*0.5*	*0*	*1*	*3*

After obtaining general information on the reaction behaviour, the conditions were screened using a 3^3^-factorial design, giving a total of 27 experiments (Table 16). The factors evaluated were initial concentration of ANI, hydrogen pressure, and temperature after 120 min of reaction with 50 mg of catalyst.

**Table 16:** Experimental design for the screening of the reaction conditions in a batch system for the amination of FUR with ANI over a Pd/ZrO_2_-TiO_2_ catalyst. Initial concentration of FUR was 0.5 mol L^-1^ and the solvent was TAA.

ID	Reaction conditions		
	P H_2_ (bar)	T ( °C)	C^0^_ANI_ (mol l^-1^)
**EXP-1**	0.5	50	0.125
**EXP-2**	0.5	50	0.25
**EXP-3**	0.5	50	0.5
**EXP-4**	0.5	75	0.125
**EXP-5**	0.5	75	0.25
**EXP-6**	0.5	75	0.5
**EXP-7**	0.5	100	0.125
**EXP-8**	0.5	100	0.25
**EXP-9**	0.5	100	0.5
**EXP-10**	1	50	0.125
**EXP-11**	1	50	0.25
**EXP-12**	1	50	0.5
**EXP-13**	1	75	0.125
**EXP-14**	1	75	0.25
**EXP-15**	1	75	0.5
**EXP-16**	1	100	0.125
**EXP-17**	1	100	0.25
**EXP-18**	1	100	0.5
**EXP-19**	2	50	0.125
**EXP-20**	2	50	0.25
**EXP-21**	2	50	0.5
**EXP-22**	2	75	0.125
**EXP-23**	2	75	0.25
**EXP-24**	2	75	0.5
**EXP-25**	2	100	0.125
**EXP-26**	2	100	0.25
**EXP-27**	2	100	0.5

For the kinetic experiments, a 20 mL SS316 autoclave reactor equipped with a PTFE liner was used following the protocol described by Ortega et al. [17] (Fig. 2). The reactions were carried out by varying one factor at a time (initial concentration of reactants, hydrogen pressure and temperature), as shown in Table 17. Samples of the liquid phase were taken at different reaction times, up to 120 min.

**Table 17:** Dynamic reaction conditions for the amination of FUR with ANI. In TAA as a solvent media and Pd/ZrO_2_-TiO_2_ as catalyst.

Exp.	Temperature (°C)	H_2_ pressure (bar)	ANI (mol l^-1^)	FUR (mol l^-1^)
1	**50**	1	0.125	0.5
2	**75**	1	0.125	0.5
3	**100**	1	0.125	0.5
4	50	**2**	0.125	0.5
5	50	**1**	0.125	0.5
6	50	**0.5**	0.125	0.5
7	50	1	**0.125**	0.5
8	50	1	**0.25**	0.5
9	50	1	**0.5**	0.5
10	50	1	0.125	**0.5**
11	50	1	0.125	**0.25**
12	50	1	0.125	**0.125**

It should be noted that all experiments carried out in the autoclave-type batch reactor were conducted in a single experimental run. However, any data showing apparent deviations from the general trends were repeated to verify their consistency before being included in the manuscript.

The products were identified by gas chromatography coupled to mass spectrometer (GC–MS). The system was configured as follows: one Rtx-5 ms capillary column (30 µm, 0.32 mm, 0.32 µm), injector temperature, ion source, and transfer line at 250 °C; an oven temperature program starting at 35 °C up to 180 °C at 5 °C min^-1^; and then from 180 °C up to 300 °C at 20 °C min^-1^. The sample volume injected was one µL in split mode (ratio 5.0), with Helium G6.0 (Indura, 99.99%) as carrier gas (pressure control mode at 10 kPa). Meanwhile, the MS module was operated in electron impact mode at 70 eV and SCAN mode (*m/z*: 2 ∼ 500).

For quantification, nonane was used as an internal standard, and all samples were analyzed on an SRI ex situ gas chromatograph (GC-model 8610) equipped with a flame ionization detector (FID) and an MTX-5 column (30 m x 0.25 mm x 0.1 µm). For the quantification of compounds that were not found commercially, the concept of effective carbon number (ECN) defined by Scanlon and Willis [18] was used. More details of the quantification method can be found in our recently published article [9].

**Fig. 3:**
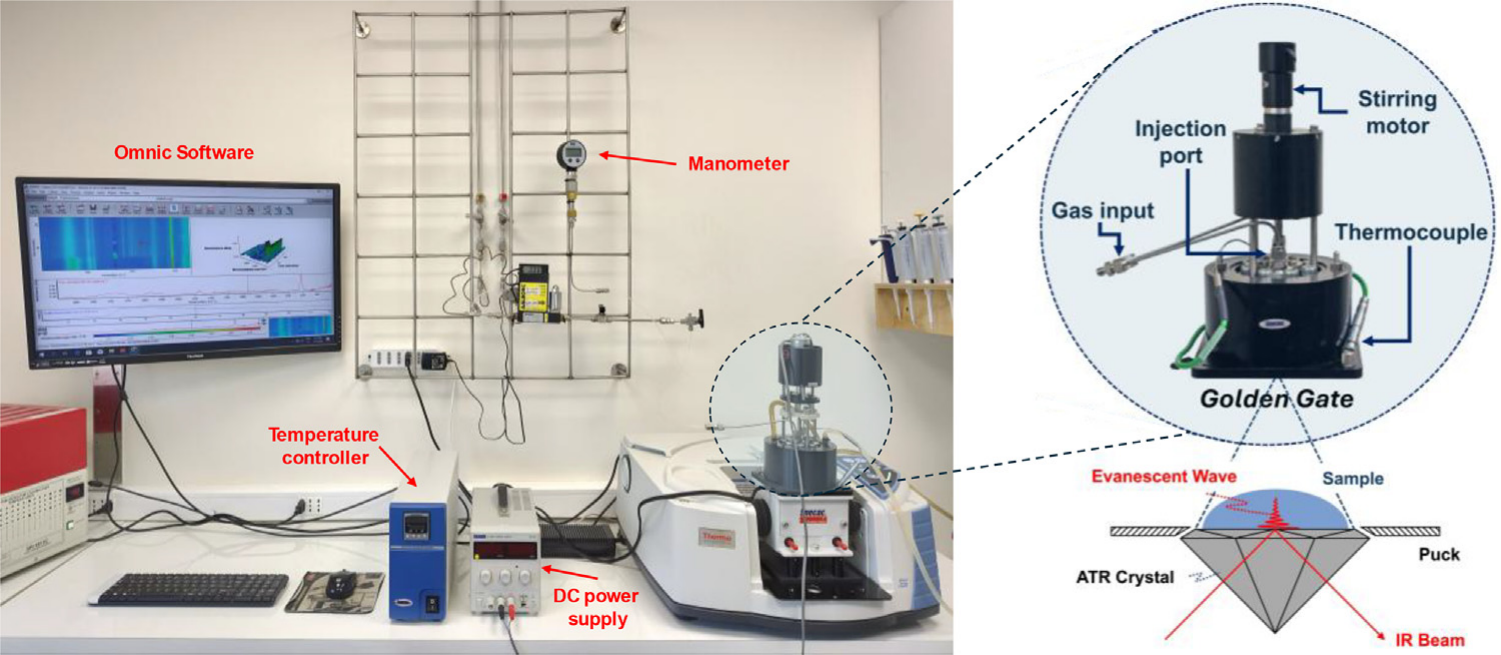
Setup of the *operando* FTIR reaction system with attenuated total reflectance (ATR) cell for measuring catalytic activity.

The experiments with the catalysts of different Zr loading (0.5, 3, 7, 10, 15, and 25 wt.% Zr on TiO_2_) were carried out under operando conditions using an attenuated total reflectance (ATR) reaction cell (Golden Gate, Specac) equipped with a diamond crystal and mounted on a Fourier transform infrared (FTIR) spectrometer (Thermo Scientific Nicolet iS20) fitted with a DTGS detector (Fig. 3). The reaction conditions were kept constants for all experiments: C^0^_ANI_ = C° _FUR_ = 0.5 mol l^-1^; T = 100 °C; P_H2_ =3 bar; t_R_ =120 min; V_R_ = 10 mL.

**Fig. 4:**
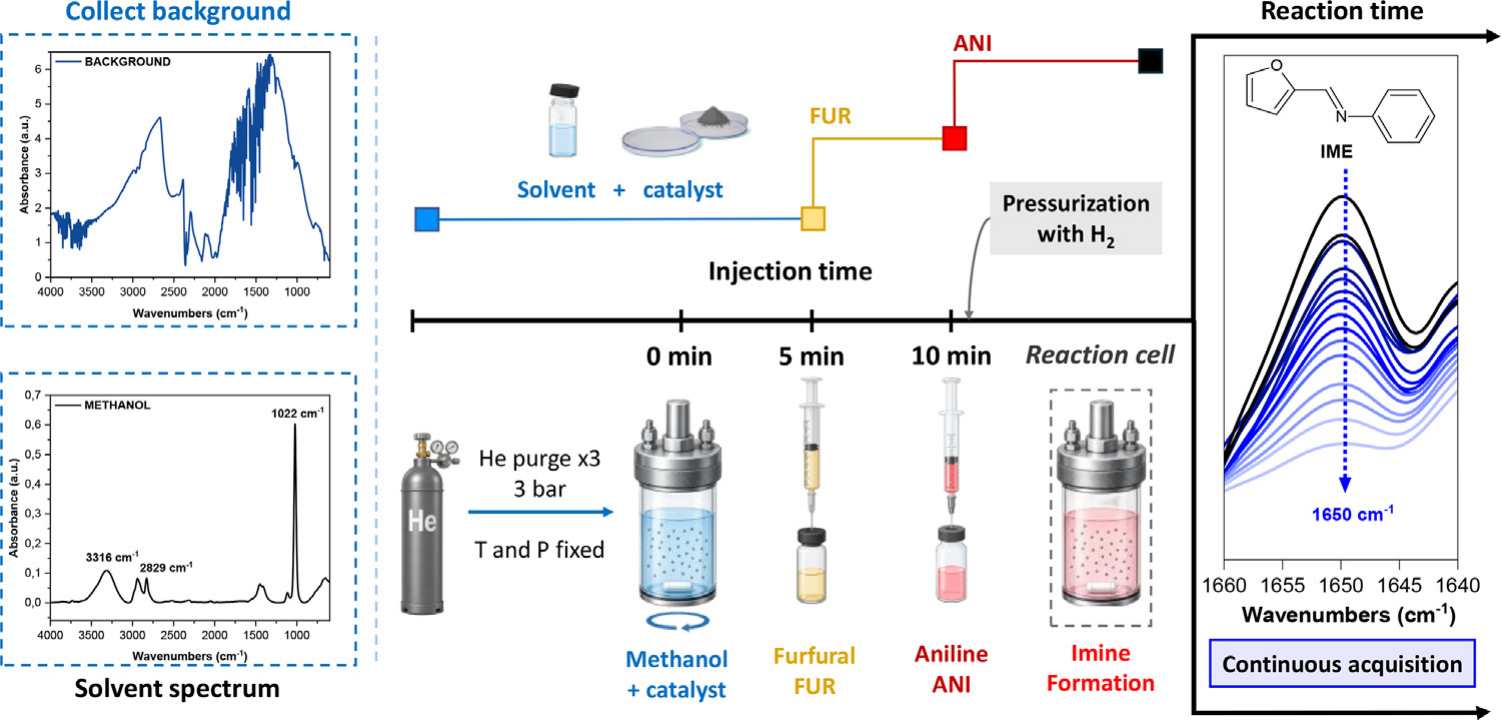
Experimental protocol for monitoring catalytic reactions in *operando* FTIR spectroscopy system.

Fig. 4 shows the experimental protocol to conduct tests on the FTIR-ATR spectroscopy system. Prior to the dynamic FTIR measurements, background spectra were recorded for the empty cell, the solvent, and the pure reagents, to correct for possible atmospheric interferences and identify any signal overlaps. Subsequently, the reactor was loaded with the solvent (methanol) and the catalyst, maintaining stirring at over 700 rpm. Once the reactor was sealed, the temperature and pressure conditions were established, initially under an inert helium atmosphere. Following this, spectrum acquisition began using the OMNIC Specta software (t = 0 min). Before the addition of the reactants, the reaction cell and gas lines were subjected to three cycles of pressurization and purging with helium at 3 bar to remove moisture and residual oxygen. Subsequently, the reaction temperature was adjusted. Then, furfural (FUR) and aniline (ANI) were injected at t = 5 min and t = 10 min, respectively, followed by pressurization with hydrogen. During the reaction, spectra were continuously acquired in the mid-infrared region, between 4000 and 600 cm^-1^, using a resolution of 4 cm^-1^ and 32 sweeps per spectrum. Finally, the spectra obtained were processed using baseline correction, multiplicative scattering correction (MSC), and normalization relative to the area of the solvent’s characteristic peak at 1022 cm^-1^.

The response variables for all activity assays were conversion, selectivity, yield, and turn over frequency (TOF) which were calculated using (1), (2), (3), (4) respectively, like those suggested by Vannice [19].

(1) $$X_{i,t}=\frac{C_{i}^{o}-C_{i,t}}{C_{i}^{o}}\;*\;100$$

(2) $$Y_{j}=\frac{C_{j}}{C_{FUR}^{0}}*100$$

(3) $$S_{j}=\frac{Y_{j}}{X_{FUR}^{0}}*100$$

(4) $$TOF=\frac{\frac{\left(dC_{FFA}\right)}{dt}}{n_{M,tot}*D}$$

**Fig. 5:**
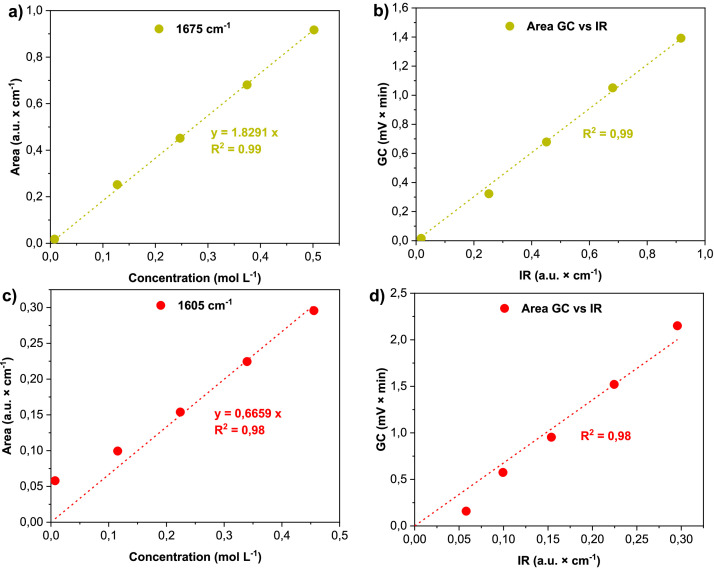
ATR-FTIR calibration curves and GC-based validation plots for furfural (a,b) and aniline (c,d) quantification.

$C_{i}^{0}$ denotes the initial concentration of reactant i, whereas $C_{i,t}$ represents its concentration at reaction time t. $C_{j}$ corresponds to the concentration of product j, $C_{FUR}^{0}$ is the initial furfural concentration in the reactor, and $X_{\text{FUR}}$is the furfural conversion. The turnover frequency (TOF, s^-1^) was obtained from the polynomial differentiation of the normalized area–time profiles associated with FFA formation, evaluated at t=0, and normalized by the amount of exposed metal according to Eq. (4). All values were determined from IR spectroscopic measurements and subsequently corroborated by GC-FID and calibrated with analytical standards as shown in Fig. 5.

## Limitations

The quantification of reaction products such as imines derived from the different N-Substrates was hampered by the unavailability of commercial standards (high purity). Given this limitation, the data presented for these products were calculated using the effective carbon number (ECN) concept*.*

## Ethics Statement

The authors state that neither humans nor animals were involved in the acquisition of the dataset. These data were obtained through experiments, so we do not present data from social networks or public databases.

## CRediT Author Statement

**Alex A. Fernández-Andrade:** Conceptualization, Formal analysis, Data organization, Writing - Original Draft. **Katherine A. Arriagada-Fuentes:** Data organization, Writing – review & editing, Methodology, Visualization. **Juan P. Parra:** Data organization, Writing – review & editing, Methodology, Visualization**. Cristian H. Campos:** Writing – review & editing, Supervision, Formal analysis, Conceptualization. **Luis E. Arteaga-Pérez:** Writing – review & editing, Supervision, Formal analysis, Project administration, Funding acquisition, Conceptualization.

## Data Availability

Mendeley DataDataset of furfural reductive amination with aniline on bifunctional Pd/ZrO2-TiO2 catalysts: Materials Characterization and Performance (Original data)

## Appendix Supplementary Materials

Image, application 1
