# Supplementary material for: Dataset of structure–activity relationships in Pd/ZrO2–TiO2 catalysts for furfural reductive amination: Batch vs Operando ATR-FTIR
Source: Data Brief. 2026 Jun 25;67:113011. doi: 10.1016/j.dib.2026.113011 (PMC13329566; doi:10.1016/j.dib.2026.113011)
Supplement: Supplementary file 1 [file mmc1.pdf]

Applied Catalysis A, General 705 (2025) 120455

Contents lists available at ScienceDirect

Applied Catalysis A, General

journal homepage: www.elsevier.com/locate/apcata

Exploring the bifunctional role of Pd/ZrO_2-TiO_2 in the production of secondary amines via reductive amination of furfural

Alex A. Fernández-Andrade [a], John A. Vergara [b], Daviel Gómez [c], Daniela González-Vera [d], Cristian H. Campos [d], Joan M. Rodríguez-Díaz [e], Luis E. Arteaga-Pérez [f,*]

[a] Laboratory of Thermal and Catalytic Processes (LPTC-UBB), Department of Process Engineering and Bioproducts, Engineering Faculty, Universidad del Bio-Bio, Concepción 4030000, Chile
[b] University of Concepción, Faculty of Chemical Sciences, Department of Physicochemistry, Edmundo Larenas 129, Concepción, Chile
[c] Instituto de Tecnología Química, Universitat Politècnica de València-Consejo Superior de Investigaciones Científicas (UPV-CSIC), Avenida de los Naranjos s/n, Valencia 46022, Spain
[d] Universidad Andres Bello sede Concepción, Facultad de Ciencias Exactas, Departamento de Ciencias Químicas, Autopista Concepción-Talcahuano Talcahuano 7100, Chile
[e] Departamento de Procesos Químicos, Biotecnología y Alimentos, Facultad de Ingeniería y Ciencias Aplicadas, Universidad Técnica de Manabí, Manabí, Ecuador
[f] Department of Chemical Engineering, Faculty of Engineering, Universidad de Concepcion, Concepción 4030000, Chile

ARTICLE INFO

Keywords:
Bifunctional catalyst
Furfurylamines
Reductive amination
Kinetic modelling

ABSTRACT

Environmental concerns and the pressing need for sustainable technologies have increased the interest in furfural (FUR) amination as an environmentally friendly alternative to current petroleum-based amination processes. Here we systematically investigate the bifunctional effect of Pd/ZrO_2-TiO_2 for the one-pot reductive amination of FUR with aniline (ANI) using H_2 as the reducing agent. The structural characterization of Pd/ZrO_2-TiO_2 indicated Ti atom substitutions by Zr, highly dispersed Zr sites, and modulated acidity. The Pd/ZrO_2-TiO_2 catalyst was active and stable for converting FUR into N-furfurylaniline (FFA) following a sequential reaction map, involving the formation of an intermediate imine (IME), with subsequent hydrogenation. The water formed in the first reaction step does not interfere in the reaction performance as confirmed by FTIR-ATR *in situ* measurements. Finally, the effectiveness of the catalyst was explained by the presence of acid sites which enhance C═O and N = H activation, with subsequent hydrogenation promoted by Pd^0 sites.

1. Introduction

The dependence of the chemical, polymers and fuel industries on crude oil, have motivated scientific interest in finding renewable and more sustainable raw materials. In this sense, lignocellulosic biomass (LCB) and its derivatives, has emerged as potential feedstocks for obtaining chemical products of high industrial value [1]. Furfural (FUR) is one of the main LCB-derived chemicals, produced industrially from maize residues. This compound has become an interesting platform for dissimilar synthetic processes, such as the production of fuel additives, polymers, tissue scaffolds and *aromatic amines* [2]. The fossil origin of aromatic amines and their demand by the refining, pesticides, and pharmaceutical industries have led to a growing interest in their production through more sustainable processes [3].

The synthesis of aromatic amines has conventionally been carried out by hydrogenation of furfural phenylhydrazone, furfural oxime, or furfuryl azides. However, these reactions require severe operational conditions and a high energy demand for product separation and purification [4]. In view of these limitations, the heterogeneously catalyzed reductive amination of FUR has emerged as a viable alternative to produce aromatic amines [5,6]. The reductive amination of FUR proceeds through two steps when aniline (ANI) serves as the nitrogen source. The first step involves the condensation of the carbonyl group (C═O) of the FUR to form furfuryl-N-phenylmethanimine; thereafter, the C═N bonds of this intermediate imine (IME) undergo hydrogenation to form N-furfurylaniline (FFA). However, during this process side reactions leading to the formation of undesired products such as furfuryl alcohol (FOL) or the over-hydrogenation of ANI, FFA, among others take place [7]. Additionally, FOL can act as a precursor of polymers or could react with FUR to produce hemiacetals and acetals, thus competing with

* Corresponding author.
E-mail address: larteaga@udec.cl (L.E. Arteaga-Pérez).

https://doi.org/10.1016/j.apcata.2025.120455
Received 23 March 2025; Received in revised form 20 June 2025; Accepted 17 July 2025
Available online 18 July 2025
0926-860X/© 2025 Elsevier B.V. All rights are reserved, including those for text and data mining, AI training, and similar technologies.

ANI for the catalytic active sites [8,9]. These side reactions cause the reduction of product selectivity as well as the deactivation of the catalyst. Saini et al. [2], suggest that metal sites with balanced hydrogenating capacity could control these selectivity-related issues.

Among the most widely used catalysts for reductive amination of FUR are metal nanoparticles such as Pt, Pd, Ni, Cu, Ru, Rh, Co, and Au [10–12], supported on acidic (Zeolite, TiO_2, Al_2O_3, Nb_2O_3) [13–15], basic (CeO_2, CaO, MgO) [11,16], amphoteric (Y_2O_3, ZrO_2) [17,18], and neutral (SiO_2, Carbon-based) supports [14,19,20]. The selection of the metal-support pair is relevant for the process as their interaction influences on the hydrogenation capacity of the active sites. For example, Xie et al. [21], demonstrated that metals with moderate H_2 adsorption capacity, such as Ru, Co, and Ni, require a basic support to provides electron density to enhance their interaction with H_2 during reductive amination. On the contrary, metals with high H_2 adsorption capacity, such as Pd, Pt, and Rh, can form secondary amines independently of their interaction with the support, causing, in some cases, over-hydrogenation of the products. The ability of metals to activate hydrogen depends on the electron density around hydrogen, so acid supports could regulate this property and, in turn, improve the selectivity of the system. To demonstrate this hypothesis, bifunctional metal catalysts (Pd, Pt, and Rh) with high H_2 activation capacity dispersed on supports of different natures have been used [22,23]. These studies confirmed that when FUR is aminated with NH_3, the metal and supports sites play a bifunctional role in the amination of FUR to primary amines [11,19]. However, this approach has not been studied in detail for the synthesis of secondary aromatic amines from FUR.

As a contribution to this knowledge gap, García-Ortiz et al. [7] evaluated the amination of FUR with ANI using Pd catalysts supported on carbon (neutral) and Al_2O_3 (acid). The results were explained by infrared spectroscopy and indicated that FUR can interact with the acidic sites of the support through the C=O and furanic ring. They proposed that the reaction take place in two stages: first the FUR interacts with ANI to form a Schiff base, which thereafter is hydrogenated into an aromatic secondary amine. Moreover, García-Ortiz et al. [7] witnessed a reduction in the selectivity to the secondary amine owing to the hydrogenation of the furanic ring. Under a similar approach, Martinez et al. [24] studied the reductive amination of FUR with ANI using SiO_2 functionalized with sulphonic acid as support for Ir, Pt, and Au. The authors highlighted that the acidic sites of the support and the nucleophilic character of the ANI favoured the formation of the IME intermediate which was explained by the protonation of the C=O in the acidic sites. This increases the electrophilic nature of the C=O, which enhances reactivity with the amino group in the formation of IME. Despite the remarkable results found so far, highly acidic supports tend to form strong metal-support interactions (SMSI) [25], leading to the reduction of the support and covering the H_2-activating metallic sites. These SMSI effects limits the hydrogenation of imine (C=N), thus hindering the selectivity to secondary amines during the reductive amination of FUR.

Despite ZrO_2-TiO_2 have shown tuneable acidic properties that agree with Tanabe`s model, there are few reports on their application for FUR amination [26]. Tanabe's model indicates that the incorporation of Zr on TiO_2 leads to a modulation of the nature and strength of the active sites as a function of the molecular proportions of Ti and Zr [27]. This balance in acidity would allow controlling the strength at which reactants, intermediates, and products adsorb on the catalytic surface, inhibiting eventual SMSI effects that affects selectivity in some traditional catalysts. In addition, this mixed oxide support has been shown to uniformly disperse nanoparticles of metals such as Pd, Ni, Co and Pt, favouring catalytic activity, mainly in the hydrogenation steps [28–30].

The prior considerations demonstrate the need for bifunctional catalysts that integrate metal and acidic sites to improve product selectivity in FUR amination. Pd supported on ZrO_2-TiO_2 may serve as a promising alternative, enabling the modulation of surface properties with the hydrogenation capacity of the metal sites to regulate its efficacy of FUR amination. To the best of our knowledge, this is the first systematic evaluation of the catalytic performance and kinetic behaviour of Pd/ZrO_2-TiO_2 in the reductive amination of furfural to secondary aromatic amines. Catalytic measurements were correlated with comprehensive physicochemical characterisation and kinetic modelling to obtain fundamental insights into the role of the catalyst's bifunctionality, reaction pathways, and mechanistic steps on product selectivity. These results contribute to the rational design of advanced catalytic materials for the valorisation of lignocellulosic biomass.

2. Materials and methods

2.1. Catalysts synthesis

Titanium dioxide (Alfa Aesar, TiO_2: CAS-1317–70–0), zirconium butoxide ($C_{16}H_{40}O_4Zr$: CAS-1071–76–7), and absolute ethanol (C_2H_6O: CAS-64–17–5), the latter provided by Sigma Aldrich®, were used for the synthesis of the ZrO_2-TiO_2 support. The support was prepared at 3 wt% Zr by a wet impregnation method. Briefly, Zr butoxide was added to a glass ballon containing 50 mL of ethanol. The solution was homogenized in an ultrasonic bath for 5 min, then TiO_2 (125–210 μm) was added; thereafter the mixture was homogenized for another 5 min. Then, the resulting suspension was stirred in a rotary evaporator for 30 min at 100 r.p.m. and 40 °C. The solid recovered, after solvent evaporation, was oven-dried (Lab Tech, LDO-150F) at 50 °C for 24 h and thermally treated using air flow (50 mL min^{-1}) at 500 °C for 2 h (2 °C min^{-1}) to obtain a mixed oxide labelled as ZrO_2-TiO_2.

Two Pd catalysts (1.5 %) supported on ZrO_2-TiO_2 and SiO_2 (as a neutral reference support) were synthetized using $PdCl_2$ (Merck, CAS-7647–10–1 >99 % purity) as the metallic precursor. The synthesis was carried out by following the incipient wetness impregnation protocol proposed by Ortega et al. [31]. Briefly, the impregnating solution was prepared by dissolving $PdCl_2$ in double distilled water, and the pH was adjusted at 1.5 with HCl (Merck, CAS-7647–01–0, 37 %). Then triethanolamine (TEA, ≥99 %, Merck, CAS-102–71–6) in a molar ratio of 4:1 (TEA:Pd) was added dropwise to the solution to enhance the metal dispersion. Then, Pd-impregnated supports (catalyst precursors) were oven-dried for 48 h. Thereafter, the catalyst precursors were calcined at the same conditions as the support, and prior to their use, they were treated under flowing H_2 (40 mL min^{-1}) (air liquid, 99.99 %) at 400 °C for 2 h according to prior temperature programmed reduction experiments (TPR-H_2).

2.2. Catalyst characterization

The catalysts were characterized by several techniques to inspect their textural, structural and surface chemical properties.

The composition of the crystalline phases of supports and catalysts were investigated by X-ray diffraction (XRD) in a Bruker D4 diffractometer with CuKα radiation (λ = 0.15418 nm) in the 2θ angular range from 3 to 90°. The results were analyzed in the X'Pert HighScore Plus software by comparing the patterns with the Inorganic Crystal Structure Database (ICSD). In addition, a Rietveld refinement was performed on all patterns to determine the relative abundance of the phases and to estimate the crystallite size more accurately. The quality of this refinement was evaluated in terms of the discrepancy factor (weighted residual error, Rwp). To determine the average size of metallic particles, transmission electron microscopy (TEM) and high-resolution TEM (HR-TEM) techniques were employed with a JEOL JEM 1200 EXII electron microscope at 120 kV and a JEOL JEM 2010 electron microscope operating at 200 kV with a resolution of 2.35 Å. The images were processed using the ImageJ software, to measure the sizes and count the Pd particles. The average particle sizes were used to calculate the Pd dispersion on the supports using the Eq. 1.

$$D = \frac{6 \quad x \quad (V_m/a_m)}{d_p} \tag{1}$$

here D is the metal dispersion, V_m is the volume occupied by an atom in the bulk metal, a_m is the area occupied by a Pd atom, d_p is the average nanoparticle size, and the number six assumes hemispherical nanoparticles.

The measurement of the interplanar distances of the crystalline structures was performed with the Gatan Microscopy Suite Software through image simplification by applying a Fourier transform [32]. The configuration of high-angle annular dark-field scanning transmission electron microscopy (STEM) and the energy-dispersive X-ray spectroscopy (EDX) were performed simultaneously for chemical analysis, using an Euro EA3000 (EuroVector) elemental analyser.

Surface area, volume, and average pore size were estimated by N_2 physisorption at 77 K in a Micromeritics Gemini VII 2390 t by fitting the BET model to the experimental data. The reducibility of the catalyst species was verified by temperature programmed reduction experiments (TPR-H_2) up to 550 °C in a Micromeritics ASAP 2010 device under 50 mL min^{-1} of 5 % v/v H_2/Ar.

The total acidity and their strength were determined by the temperature-programmed desorption of ammonia (TPD-NH_3) technique. The experiments were carried out using the 3FLEX (Micromeritics) apparatus equipped with a TCD combined with a mass spectrometer (Pfeiffer Vacuum OmniStar GSD 320). About 100 mg of sample was pretreated at 350 °C (20 °C min^{-1}) for 30 min in a continuous flow of He. Thereafter, the catalyst was cooled down in He to 100 °C and then NH_3 was fed by pulses at an interval of 10 min (loop of 0.5 cm^3) in a continuous flow of 100 mL min^{-1} of He. After saturating the surface with NH_3, it was kept at 100 °C for 30 min in He (100 mL min^{-1}) to remove the weakly adsorbed NH_3 and stabilize the baseline. Finally, the sample was heated in He up to 750 °C with a ramp of 10 °C min^{-1}. The signals of NH_3, H_2O, N_2 and N_2O were recorded using an online mass spectrometer following specific fragments, *m/z*: 16, 18, 28 and 44, respectively. Desorption profiles and acid site density were normalized per gram of catalyst.

The type of acidity of the support was studied by infrared pyridine adsorption (IR-Pyr). The sample wafer was pretreated at 400 °C for 12 h in vacuum conditions, then cooled at 150 °C and the reference spectrum was taken. Subsequently the pyridine fed for 10 min. The measurements were carried out at three different temperatures (150, 250 and 350 °C) after 30 min at each temperature in vacuum conditions.

The chemical state of Pd was identified by X-ray photoelectron spectroscopy (XPS) in an instrument with a PHOIBOS 150 MCD 9 analyser from SPECS and a non-monochromatic Alkα X-ray energy of 1486.60 eV. The energy was corrected using the binding energy 103.4 eV of the Si 2p component for Pd/SiO_2 and 459.0 eV for the Ti 2p of Pd/ZrO_2–TiO_2. The data were processed with CasaXPS software (Casa Software Ltd) and using the NIST library [33]. The spectra of the materials were collected after *ex situ* reduction at 500 °C for 2 h under H_2.

2.3. Catalytic activity tests

Control reactions with FUR (Sigma Aldrich, CAS: 98–01–1), ANI (Sigma Aldrich, CAS: 62–53–3), and H_2 (Air Liquide, 99.99 %) in tert-amyl alcohol (Merck, CAS: 75–85–4) were carried out in a 3 mL reinforced glass autoclave reactor. The rector was placed in a Reacti-Therm™ system (Thermofisher, USA) equipped with an external temperature sensor and magnetic stirring (at 900 r.p.m.). In a typical experiment, the reactor was loaded with 0.5 mol L^{-1} FUR, 0.5 mol L^{-1} ANI, and the volumetric difference with tert-amyl alcohol. The system was pressurized with 3 bar of H_2, and the reaction was conducted at 100 °C for 120 min. Under these conditions, different reaction systems were evaluated, including homogeneous reactions (without catalyst), direct reductive amination of FUR, and hydrogenation of FUR to FOL.

After the preliminary assays, a systematic study on the effect of the reaction conditions such as amine concentration (C^0_{ANI} = 0.125, 0.25, and 0.5 mol L^{-1}), temperature (50, 75, and 100 °C), and H_2 pressure (0.5, 1, and 2 bar) was carried out. The study followed a factorial design of 3^3, which can be found in Table S1. Measurements were carried out during 120 min using a fixed initial concentration of FUR (C^0_{FUR} = 0.5 mol L^{-1}), and 50 mg of catalyst (Eqv. Substrate/Catalyst = 212 mol FUR/mol Pd).

After exploring the reaction conditions, we performed a preliminary kinetic analysis of the FUR reductive amination. These measurements were carried out in a 20 mL SS316 autoclave reactor equipped with a PTFE liner, pressurization gas lines and a sampling port. The reaction conditions were evaluated in the same range as mentioned above for the experimental design; although in this case we varied the initial concentration of FUR ($C^0{}_{FUR}$ = 0.125, 0.25, and 0.5 mol L^{-1}). Samples of the liquid phase were taken at regular reaction times up to 120 min. The presence of external and internal mass transfer limitations were ruled-out to guarantee that the reactions proceeded under a kinetically controlled regime (See Table S2) [34].

2.4. Product identification and quantification

The reaction products were identified in a PerkinElmer Clarus 690 gas chromatograph connected to a PerkinElmer Clarus SQ-8T mass spectrometry detector (GC-MS). The system was configured as follows: one Rtx-5 ms capillary column (30 µm, 0.32 mm, 0.32 µm), injector temperature, ion source, and transfer line at 250 °C; an oven temperature program starting at 35 °C follow by a heating up to 180 °C at 5 °C min^{-1}; and then from 180 °C to 300 °C at 20 °C min^{-1}. The sample volume injected was one µL in split mode (ratio 5.0), with Helium G6.0 (Indura, 99.99 %) as carrier gas (pressure control mode at 10 kPa). Meanwhile, the MS module was operated in electron impact mode at 70 eV and SCAN mode (*m/z*: 2 ~ 500). A typical chromatogram with the corresponding ionization patterns for the reactants and products is provided in Table S3.

For quantification, nonane was used as an internal standard, and all samples were analysed on an SRI ex situ gas chromatograph (GC-model 8610) equipped with a flame ionization detector (FID) and an MTX-5 column (30 m x 0.25 mm×0.1 µm). Compounds that were not found commercially in pure form, were quantified using the concept of effective carbon number defined by Scanlon and Willis [35] and replicated by Bernt et al. [36]. Details of these calculations are reported in the Supplementary Material (Table S4).

The response variables for all activity assays were conversion, selectivity, and yield, which were calculated using Eqs. 2, 3, and 4, respectively [34].

$$X_{i,t} = \frac{C_i^0 - C_{i,t}}{C_i^0} \quad (2)$$

$$S_i = \frac{n_i}{n_t} \quad (3)$$

$$Y = \frac{n_i}{n_x} \quad (4)$$

Where C_i^0 is the initial concentration of reactant i, $C_{i,t}$ t is the concentration of reactant i at time t, n_i is the moles of product i, n_t is the total moles of products formed, and n_x is the moles of FUR converted. The formation rates (r_i) were calculated by polynomial differentiation of FFA concentration vs time profiles (Figure S1) evaluated at zero, as shown in Eq. 5, like previous work of our group [37].

$$r_{FFA} = \left[\left(\frac{1}{w_{cat}}\right)\left(\frac{dC_i}{dt}\right)\right]_{t=0} \quad (5)$$

Where r_i is the rate of FFA formation (mmol h^{-1} g^{-1}), w_{cat} is the mass of catalyst (g), C_i is the FFA concentration (mol L^{-1}).

3. Results and discussion

3.1. Structural and morphological properties

The XRD patterns of the TiO_2, ZrO_2-TiO_2 and Pd/ZrO_2-TiO_2 are shown in Fig. 1. All the patterns present intense and well-defined signals owing to the crystallinity and fine grain size of the materials. The diffraction peaks at 2θ = 25.1°, 37.4°, 47.8°, 53.3°, 54.7°, 62.2°, 68.1°, 69.8°, 74.4° and 82.0° are attributed to the (011), (004), (020), (015), (121), (024), (116), (220), (125) and (224) planes of the anatase TiO_2 phase with a tetragonal crystalline system (ICSD 9855) [29]. The broad signal close to ~15° observed in all the materials can be ascribed to amorphous phase of TiO_2. Moreover, in the case of ZrO_2-TiO_2 and Pd/ZrO_2-TiO_2 the results suggest that some Zr atoms replaced the positions of Ti atoms, increasing the unit cell parameters, which could be explained due to the larger unit cell parameters of zirconium. Therefore, the diffraction peak of Zr-substituted system shifted to a higher angles from 25.14° for pure TiO_2 to 25.21° for ZrO_2-TiO_2 (shown in zoom Fig. 1), indicating that the Zr dopant does not affect the crystallinity of TiO_2, but ZrO_2 may be formed in the titanium matrix [38,39]. These results were confirmed with the Rietveld refinement (Figure S2), which also helped to calculate the crystallite size more accurately using the Scherrer equation [40]. The crystallite sizes were ~11 nm and ~15 nm for anatase TiO_2 and ZrO_2-TiO_2, respectively. The increase in crystallite size for anatase following Zr impregnation aligned with the cell parameters and the width of the XRD data.

Interestingly, no diffraction peaks associated to Zr or Zr-oxides are detected, which could be explained by a good dispersion of Zr on TiO_2 as well as by an overlapping of some diffraction signals characteristic of Zr. Likewise, no Pd or PdO peaks are observed, probably due to the low Pd loading (~ 1.5 wt%) in the catalyst. These results also suggest the presence of well-dispersed Pd nanoparticles (undetectable by XRD) on the ZrO_2-TiO_2 support, which was further confirmed by transmission electron microscopy images (Figure S3).

The Pd/SiO_2 catalyst prepared for comparison purpose (Figure S4), shows broad diffraction peaks representative of the amorphous structure of SiO_2 [31], and a weak peak at 2θ ~39° attributed to the Pd nanoparticles (ICSD 98–004–1517), this diffraction peak also may be associated with the low metal loading and the good dispersion of the nanoparticles [28,41].

The crystal structure and surface nature of the Pd/ZrO_2-TiO_2 catalyst was confirmed by high resolution TEM (Fig. 2). The analysis of lattice spacing reveals the presence of anatase-TiO_2 (distorted by the Zr presence) and metallic Pd, characterized by a lattice spacing of 0.355 nm and 0.268 nm, correspond to the (011) and (111) planes, respectively, in agreement with the crystallographic references ICSD 98–000–9855 of anatase TiO_2 and 98–004–1517 of Pd^0. The above structural analysis confirms the increased unit cell parameter and the successful preparation of Pd/ZrO_2-TiO_2 catalyst.

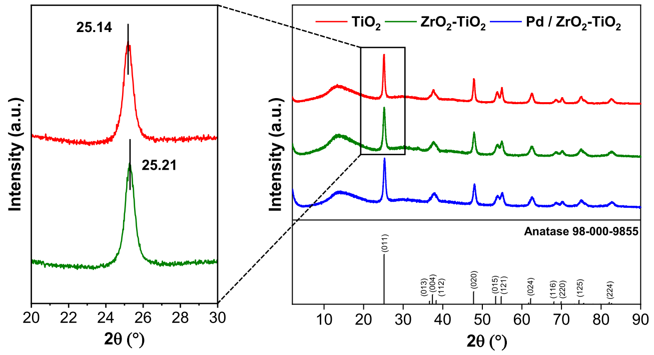

Fig. 1. X-ray diffraction (XRD) patterns for the TiO_2 based materials (TiO_2, ZrO_2-TiO_2 and Pd/ZrO_2-TiO_2 after reduction *ex situ* at 500°C).

Figure S3 shows the particle size distribution (PSD) histograms for Pd/SiO_2 and Pd/ZrO_2-TiO_2. The PSD was obtained by counting approximately 200 particles for each catalyst. The histograms confirm the presence of a narrow size distribution of Pd particles regardless of the support used. In the case of Pd/SiO_2, the Pd particles presented a normal distribution centred at 6.7 nm ± 2.1 (Figure S3-a), and for Pd/ZrO_2-TiO_2, they were centred at 7.4 nm ± 2.0 (Figure S3-b).

Figures S5 and S6 show HR-TEM images acquired at different locations on the Pd/SiO_2 and Pd/ZrO_2-TiO_2 catalysts, respectively. The presence of small Pd nanoparticles was confirmed through contrast differences with the support and by STEM-EDX measurements. Figures S7 and S8 clearly illustrate the distribution of Pd, Si and O of Pd/SiO_2 control system, and Pd, Ti, O and Zr of the Pd/ZrO_2-TiO_2 catalysts. Nevertheless, this result should be interpreted with caution because the Pd loading (~ 1.5 %) is at the detection limit of the equipment.

Therefore, similar particle sizes lead to similar dispersions of 16.6 % for Pd/SiO_2 (0.023 mmol Pd exposed/g cat.) and 15.0 % for Pd/ZrO_2-TiO_2 (0.021 mmol Pd exposed/g cat.). For a more precise study, the structural effects between catalysts can be ruled out and study only the role of acidic support in the reaction.

3.2. Surface properties of catalysts

The N_2 adsorption-desorption isotherms presented a hysteresis loop that closes at approximately $p/p^0 = 0.5$ due to capillary condensation inside the pores (Fig. 3a). This behaviour suggests that the catalyst surface contains mesopores over 4 nm and significant material-adsorbate interactions, which correspond to IV(a) isotherms according to IUPAC [42]. The hysteresis loop for Pd/SiO_2 (H1 type isotherm) indicates the presence of uniform cylindrical pores characteristic of synthetic materials such as SiO_2. Whereas ZrO_2-TiO_2 and Pd/ZrO_2-TiO_2 presented hysteresis closer to H3, suggesting non-uniform slit-like pores [43]. In terms of surface area, the Pd/SiO_2 catalyst was higher (220 m^2 g^{-1}) than Pd/ZrO_2-TiO_2 (101 m^2 g^{-1}) but lower than pure SiO_2 (Table 1). Similarly, the surface area of ZrO_2-TiO_2 (130 m^2 g^{-1}) was reduced by Pd incorporation, which is consistent with the pore volume (Fig. 3b) and may be associated with Pd deposition inside the pores.

The reducibility of the calcined catalysts was investigated by TPR-H_2 (Fig. 3c). The results showed strong reduction signals at low temperatures for both Pd/ZrO_2-TiO_2 and Pd/SiO_2, which are attributed to the reduction of the Pd^{+2} species to Pd^0 [44]. In the first case (Pd/ZrO_2--TiO_2), a sharp peak was observed at 63 °C, while in the Pd/SiO_2 catalyst, the peak is broader and shifted to 90 °C. Despite the position of these peaks suggest different interactions between Pd with the supports, the similarity in the TPR-H_2 profiles indicates that both catalysts can be reduced under equivalent conditions.

The total acidity and acid strength distribution of the Pd/ZrO_2-TiO_2 catalyst was determined from the ammonia desorption profile (NH_3-TPD) shown in Fig. 3d. In addition, the MS signals (*m/z*) at the outlet of the system were followed to discard desorption signals from species such as H_2O, N_2, and N_2O, which could indicate interactions and decomposition of NH_3 over the support (Figure S9a-c). The total acidity estimated for Pd/ZrO_2-TiO_2 was 193 μmol NH_3 g^{-1} with an acid density of 1.48 μmol NH_3 m^{-2} given mainly by Lewis's acid sites according to FTIR pyridine adsorption experiments (Figure S10). The desorption temperature of ammonia depends on the strength at which it adsorbs on the acid site, so the surface acidity can be classified as weak (100–300 °C), medium (300–500°C), and strong (above 500 °C) [45]. Therefore, the acidic surface of Pd/ZrO_2-TiO_2 was made up of 92 μmol NH3 g^{-1} of weak sites, 75 μmol NH3 g^{-1} of medium strength and 26 μmol NH3 g^{-1} of strong acidity. These acidic properties differ from those reported for similar mixed oxides [28,46], probably due to an effective dispersion of Zr on TiO_2, along with the formation of Zr-O-Ti bonds, which modulate acidity in accordance with Tanabe's model [26]. Moreover, with the incorporation of Zr, the strength of the sites was modified by increasing mainly the proportion of strong acidic sites (Figure S9-b) with respect to

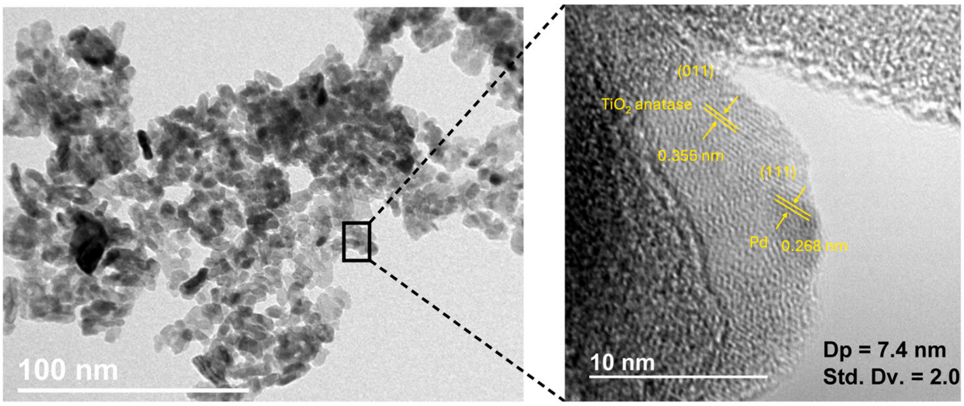

Fig. 2. TEM and HR-TEM (zoom) images of *ex situ* reduced Pd/ZrO_2-TiO_2 catalyst. Additional images are shown in supplementary information (Figs. S5 and S6).

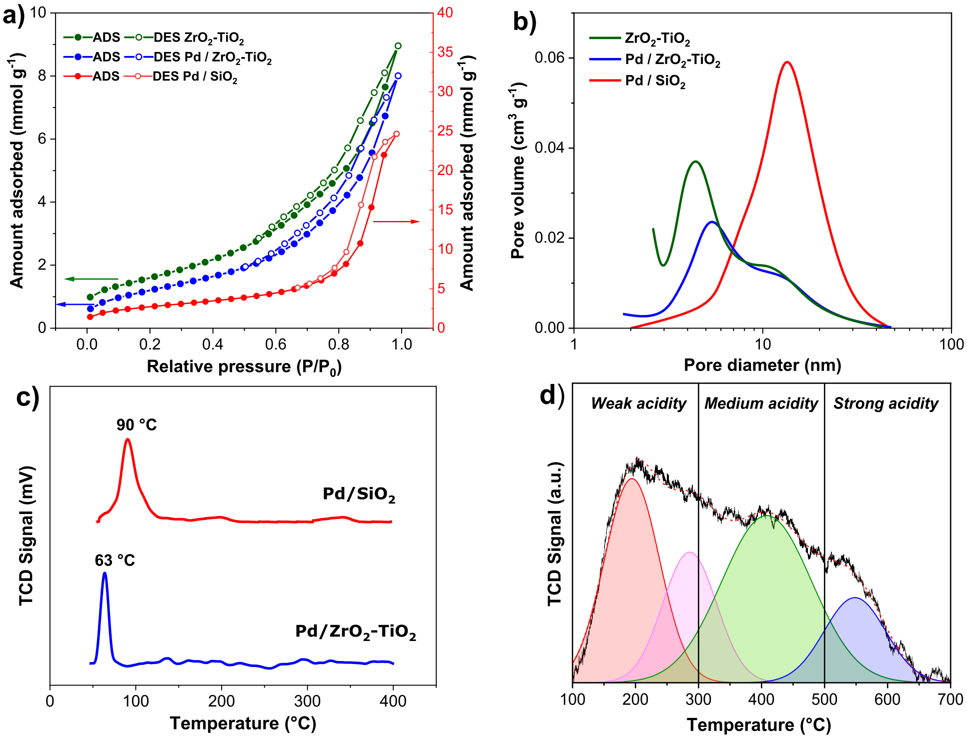

Fig. 3. Surface characterization of catalysts. a) N_2-Physisorption isotherms, b) pore size distributions, c) temperature programmed reduction profiles, and d) temperature programmed desorption of ammonia profile for Pd/ZrO_2-TiO_2.

Table 1
Textural properties of supports and catalysts.

Supports/Catalysts	S_{BET} ($m^2\ g^{-1}$)	V_p ($cm^3\ g^{-1}$)	Pore diameter (nm)
TiO_2	147	0.30	9.9
ZrO_2-TiO_2	130	0.26	9.2
Pd/ZrO_2-TiO_2	101	0.23	8.6
SiO_2	251	0.93	16.4
Pd/SiO_2	220	0.76	13.8

the pure TiO_2 (Figure S9-a), while the impregnation of Pd nanoparticles slightly modified the density of strong of medium-strength acid sites. For the Pd/SiO_2 support, the NH_3-TPD profile (not shown) confirms a negligible density of acid sites with 0.0028 μmol NH_3 m^{-2}, given by weak acidity, consistent with a previous study by our group [31].

The nature of the acid sites (Lewis and Brønsted) was studied by pyridine adsorption/desorption spectroscopy at 150, 250 and 350 °C (Figure S10). In the spectra of pure TiO_2 (Figure S10-a), ZrO_2-TiO_2 (Figure S10-b) and the Pd/ZrO_2-TiO_2 catalyst (Figure S10-c), well-defined bands corresponding to pyridine adsorbed on the Lewis acid sites are observed around 1445 cm^{-1}, 1575 cm^{-1} and 1605 cm^{-1}. The band at about 1492 cm^{-1} encloses the contribution of both Lewis and Brønsted sites. However, the absence of the adsorption band at 1540 cm^{-1} suggests that the materials do not have a significant presence of Brønsted acid sites. With the incorporation of Pd and Zr on TiO_2, the acidity of TiO_2 is modulated showing a shift towards stronger Lewis sites.

On the other hand, the chemical state of Pd and O on the surfaces of the Pd/SiO_2 and Pd/ZrO_2-TiO_2 catalysts was studied by X-ray photoelectron spectroscopy (Fig. 4). The core level of Pd 3d is shown in Fig. 4a. Spectral deconvolution of Pd $3d_{5/2}$ suggests the presence of two components on Pd/SiO_2 and Pd/ZrO_2-TiO_2 at 335.4 and 336.9 eV corresponding to Pd^0 and Pd^{2+} respectively [47,48]. The result confirms the coexistence of Pd^0 and PdO in both materials, therefore, there is no

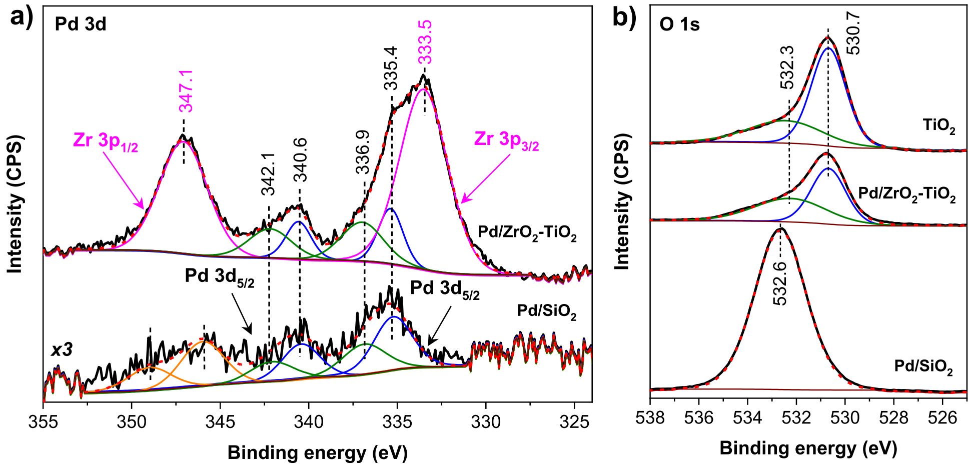

Fig. 4. XPS spectra of core levels a) Pd 3d and b) O 1 s of the *ex situ* reduced Pd/SiO_2 and Pd/ZrO_2-TiO_2 catalysts, with the reference TiO_2 support. The contribution of the core level Zr 3p was observed in the energy range of Pd 3d.

significant effect regarding the role of the support in the stabilization of different oxidation states of Pd. On the other hand, Fig. 4b shows the O 1 s core level of pure TiO_2, Pd/ZrO_2-TiO_2 and Pd/SiO_2. The silica-supported system shows a main component at 532.6 eV from the lattice oxygen on the support. The photoelectron spectra of TiO_2 and Pd/ZrO_2-TiO_2 show mainly two components. The TiO_2 support show two signals at 530.7 eV and 532.3 eV that can be attributed to the Ti-O lattices oxygens and weakly adsorbed species or hydroxyls (OH) on the surface, respectively [48,49]. The Pd/ZrO_2-TiO_2 catalyst evidence a positive shift to higher binding energies associated with the lattice oxygen of the Ti/Zr-O and surface hydroxyl groups (Ti/Zr-OH), which are in close agreement with a literature assignments for ZrO_2-TiO_2 systems [50]. This result aligns with the XRD and HR-TEM results, confirming the Zr incorporation into the titania lattice showing a strong electronic interaction by change in the surface states of the catalysts.

Previous characterizations demonstrate the textural and structural similarities of Pd-sites in Pd/ZrO_2-TiO_2 and Pd/SiO_2. Accordingly, the effectivity of these materials to catalyse FUR reductive amination will be inspected, to unravel if the nature and density of support́s acid sites provide any tuning effect in the process performance.

3.3. Preliminary experiments on catalytic activity

The catalytic performance of Pd/SiO_2 (neutral support) and Pd/ZrO_2-TiO_2 (acid support) was preliminary evaluated using nominal reaction conditions as those reported in previous papers [2,7]. Initially, control experiments were carried out to (i) study the non-catalytic reaction, (ii) probe the effect of the support on the amination of FUR, and (iii) verify the feasibility of FUR hydrogenation over Pd/ZrO_2-TiO_2 and Pd/SiO_2. The results were assessed using quantitative descriptors such as FUR conversion, and product yields (Table 2).

The direct reductive amination of FUR was investigated under non-catalytic conditions (Exp 1) as a homogeneous phase control reaction. The results show that the IME is formed in homogeneous phase with a relatively high FUR conversion (65.5 %). However, the formation of secondary amine under non-catalytic conditions was not verified, which indicates that metal and/or support sites are required for activating the hydrogen involved in the reduction of the C═N bond of IME [24,51]. To further test this theory, we replicated the homogeneous reaction at 10, 30, and 40 °C. The enthalpy (ΔH) and entropy (ΔS) results suggest that the reaction is spontaneous at low temperatures (Table S5) with FUR conversions up to 92 % (Figure S11). Moreover, the equilibrium

Table 2
Exploratory furfural amination and hydrogenation reactions. The reaction conditions were: $C^0_{ANI} = C^0_{FUR} = 0.5$ mol L^{-1}; T = 100 °C and P_{H2} = 3 bar; t_R = 180 min; substrate/catalyst molar ratio = 212 and V_R= 3 mL.

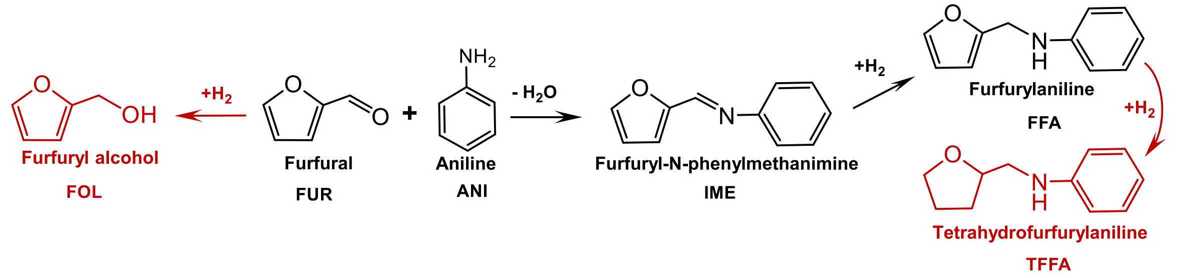

Exp.	Catalysts	X_{FUR} (%)	Y_{IME} (%)	Y_{FFA} (%)	Y_{TFFA} (%)	Y_{FOL} (%)
1	Non-catalytic	65.5	63.5	0	0	0
2	ZrO_2-TiO_2	85.7	84.3	0	0	0
3	Pd/ZrO_2-TiO_2	99.6	65.7	19.7	13.9	0
4	Pd/SiO_2	90.5	88.6	1.3	0.54	0.04
5	*Pd/ZrO_2-TiO_2	35.4	0	0	0	34.2
6	*Pd/SiO_2	19.6	0	0	0	19.6

Carbon balance for the reductive amination of furfural: 93 %
* Hydrogenation reactions of FUR (aniline-free)

calculations for this reaction were implemented in Aspen Plus v14. To implement the calculations, the IME and the secondary amines were defined according to their molecular structures, while transport and thermodynamic properties were estimated using the ThermoData Engine of the NIST database. Thereafter, the Non-Random Two Liquids model (NRTL) was used to estimate the thermodynamic properties of the species involved. Finally, a REquil model was used to study the equilibrium of FUR amination with ANI. The results indicate a FUR conversion of 94 % under the same reaction conditions as mentioned above, thus theoretically confirming the feasibility of FUR condensation under non-catalytic conditions (Figure S11).

The results for the amination of FUR over support materials evidenced that, in the presence of acidic support (Exp. 2), the reactivity of the system increases. In this case, the conversion of FUR increased to 85.7 %, probably due to the participation of the acidic sites of the support in the activation of the carbonyl bond (C=O), which enhances its electrophilic nature [52]. This makes it more susceptible to nucleophilic attack by ANI to form the IME intermediary via condensation, consistent with that reported by Martinez et al. [24]. In addition, no secondary amines or furfuryl alcohol (FOL) were formed in the absence of Pd, confirming the metal site role in the activation of H_2 and the consequent reduction effect over IME intermediary. According to Pang et al. [44], $Pd^{\delta+}$ species participate in the adsorption of substrates and intermediates, even in the absence of acid sites on the support, but Pd^0 sites are required to dissociate the hydrogen involved in the reduction steps, as observed in exp 3 and 4.

In Exps. 3 and 4, the conversion of FUR for both catalysts were higher than 90 %. However, the yield towards FFA for Pd/ZrO_2-TiO_2 (19.7 %) was significatively higher than for Pd/SiO_2 (1.3 %). Moreover, the 13.9 % yield of tetrahydrofurfurylaniline (TFFA) obtained for Pd/ZrO_2-TiO_2, confirms that the furanic ring in FFA is over-hydrogenated in the presence of acid sites. The hydrogenating capacity of Pd/ZrO_2-TiO_2 can be explained by a strong interaction of FFA with the support sites, and by the spill-over of H enhanced by the presence of acid sites nearby Pd^0 [7].

The differences in the hydrogenation capacity of Pd/ZrO_2-TiO_2 and Pd/SiO_2 were inspected by performing the reactions in absence of ANI (Exp. 5 and 6). The results show higher activity of the Pd/ZrO_2-TiO_2 catalyst to hydrogenate FUR, leading to 35.4 % conversion which is approximately 1.8 times higher than that obtained for Pd/SiO_2. In terms of yields, the evident difference in FOL formation between these catalysts strengthens the hypothesis of C=O activation of FUR occurs over acid sites. This interaction of C=O with the surface would facilitate its hydrogenation assisted by Pd^0 metal sites [53,54]. Although these results demonstrate that it is feasible to hydrogenate the C=O of FUR under the conditions studied, the absence of FOL in the amination products suggests a selective hydrogenation of C=N bonds rather than C=O adsorbed on the support.

3.3.1. *Effect of water on FFA formation*

Water is one of the products released during the first amination step (IME formation) and could play an important role in the equilibrium and kinetics of the reaction. In addition, it has an affinity for acid sites, which could modify its nature and strength under reaction conditions. For this reason, here we have preliminary addressed the implications of water in the formation of FFA using a Fourier Transform Infrared coupled Attenuated Total Reflectance (FTIR-ATR) spectroscopy cell with DTG detector. For this purpose, dynamic experiments were carried out by injecting water in an amount stoichiometrically equivalent to that generated during IME formation. First, reactions were carried out without catalysts to observe the effect of water in the homogeneous phase and then with catalyst to rule out a possible interaction with the catalytic surface (Table S6).

Figs. 5a and 5b show the spectral regions identified to follow the evolution of the compounds during the reaction. In the first case, the 1655–1645 cm^{-1} region was identified as a characteristic vibration of the C=N bond of the IME, which decreases in absorbance with time and reflects its consumption to form FFA. Meanwhile, the band in the 1240–1225 cm^{-1} region represents the vibration of the C-N bond of the FFA, which increases in absorbance with respect to time because of an increase in product concentration [55]. With the identification of the

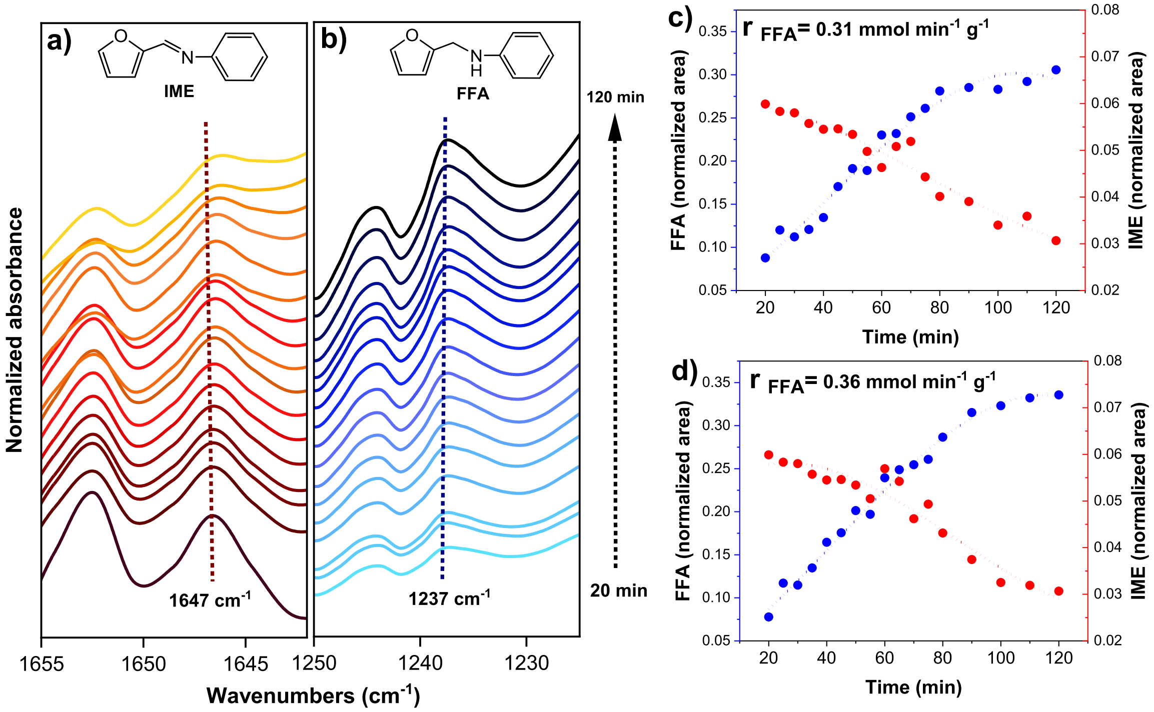

Fig. 5. Effect of water on FFA formation. a) spectral region of IME over time, b) spectral region of FFA over time. Area profiles for the catalytic reaction c) with water injection and d) without water injection. Reaction conditions: $C^0_{ANI} = C^0_{FUR} = 0.5$ mol L^{-1}, T = 100 °C, P_{H2} = 3 bar, and V_R= 15 mL, and substrate/catalyst Pd/ZrO_2-TiO_2 molar ratio = 567.

bands, the areas were calculated and normalized to the largest peak of the solvent (938 cm^{-1}). In this way, area profiles as a function of time were constructed for the catalytic reactions with and without water injection, which are presented in Figs. 5c and 5d, respectively.

The concentration profiles show quite similar trends for both catalytic conditions, where water seems not to affect FFA formation at the reaction conditions used. This first approximation is strengthened by observing the closeness of the FFA formation rates with values of 0.31 mmol min^{-1} g^{-1} for the reaction with water injection into the system and 0.36 mmol min^{-1} g^{-1} when no water was injected. This behaviour suggests that an eventual interaction of water with the catalytic surface does not inhibit the formation of the desired product. This observation suggest that the acid sites of the support could be interacting with the C═N imine group rather than hydrating, which favours the reaction towards FFA formation, as confirmed by the yield values (Table S6). In the non-catalytic experiments, the behaviour was similar, and the selectivity values did not change with the injection of water into the system.

The result from these preliminary assays indicates that there is a bifunctionality between Pd and ZrO_2-TiO_2, which allows controlling reactivity and selectivity of FUR reductive amination. In the following sections we analysed the behaviour of this bifunctional catalyst under different reaction conditions to propose a plausible reaction pathway along with a preliminary kinetic interpretation of the results.

3.4. Proposal of the reaction scheme

To propose a preliminary reaction scheme consistent with the reductive amination of FUR, a first and second range DelPlot analysis was performed (Fig. 6). The DelPlot analysis, allows identifying products formed in one-step reactions (primary products) or in sequential reactions of two or more steps (second range) [56].

According to the first rank profile (Fig. 6a), the IME has a finite Y-intercept at 0- time, which is characteristic of a primary product and suggests that the reaction between FUR and ANI is carried out at a high rate. Meanwhile, the Y/X values for the secondary amines (FFA and TFFA) intercepts the Y-axis in zero, indicating that both products are of higher order. In the second-order profile (Fig. 6b), the Y/X^2 ratio cuts into finite Y for FFA, confirming its formation as a secondary product. Whereas, for TFFA, its interception is zero, suggesting that it is a product formed in more than two reaction steps. These results correspond with our observations during preliminary assays, where secondary amines are formed in a second reaction step catalysed by metal and acid sites. In the case of FOL, DelPlot profiles are not presented because it was not formed during the FUR amination reactions. However, according to the results in Table 2, it can only be formed from FUR, so it is considered a primary product. With the consistency between the results of the control experiments and the DelPLot analysis of the system, the reaction scheme could be preliminarily structured into four possible main steps or stages (Fig. 6c).

In the first stage (I), FUR reacts with ANI by nucleophilic attack to form the IME intermediate, producing water in an apparently non-catalytic step. Then, in the second stage (II) of the main pathway (blue arrows), the IME is reduced to the secondary amine (FFA) with the H^+ activated over Pd^0 sites as previously reported for similar reactions [44,57]. The possible strong adsorption of FFA on the catalytic sites

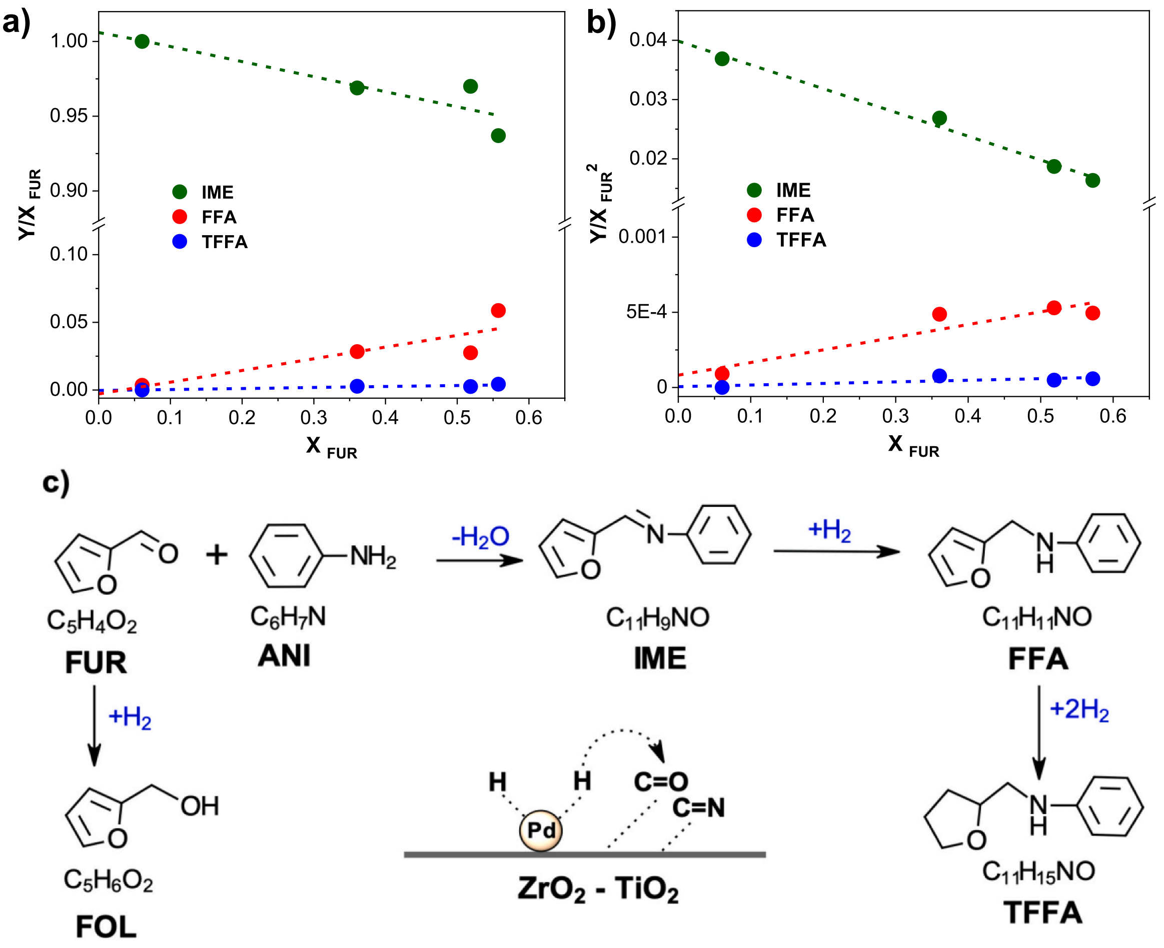

Fig. 6. DelPlot profiles of a) First range and b) second range for the amination products of FUR with Pd/ZrO_2-TiO_2 catalyst. c) Preliminary reaction scheme. C^0_{ANI} = 0.125 mol L^{-1}; C^0_{FUR} = 0.25 mol L^{-1}; T = 50 °C and P_{H2} = 1 bar; t = 120 min and substrate/catalyst molar ratio = 567.

leads to a next stage (III), where FFA is hydrogenated through the furanic ring to form a more stable secondary amine (TFFA). This last step has not been mentioned in detail by other authors, but the hypothesis agrees with the results found here on the feasibility of the reaction. Finally, in a parallel catalytic reaction (IV) to the IME formation, the carbonyl group (C=O) of FUR could be hydrogenated to form FOL. However, this reaction did not take place under the conditions used here, probably because the rate at which the IME is formed is higher, which favours the hydrogenation of the C=N bond instead of the C=O bond.

3.5. *Effect of the initial concentration of the reactants*

Studying the effect of the initial concentration (C^0) of the reagents in catalytic reactions is crucial. It provides valuable insights on the involvement of the species in the reaction mechanism, as well as information on conversion, product selectivity, as well as initial reaction rates [34]. Figs. 7, 8, and 9 demonstrate the impact of the initial concentrations of ANI, FUR, and hydrogen on the reductive amination of FUR. In addition, the reaction rates were corrected by applying the approach to equilibrium concept (Eq. 6). The equilibrium constants (K_{Eq}) were estimated at different temperatures for the FUR amination with ANI to yield FFA (Tables S5 and S7). The calculations were implemented in Aspen Plus v14.0 using a REquil reaction block and following the procedures described in Section 3.3.

$$\eta = \frac{[FFA]}{[FUR][ANI]} \bullet \frac{1}{K_{Eq}} \tag{6}$$

Fig. 7a shows a positive effect of the initial concentration of ANI on the conversion of FUR, which increases from 23.9 % (at C^0_{ANI} = 0.125 mol L^{-1}) to 87.7 % (at C^0_{ANI} = 0.5 mol L^{-1}). The ANI conversion was above 98 % in all cases, and IME formation takes place at an approximate stoichiometric ratio of 1.1: 1 (ANI: FUR), which is consistent with the step I in the proposed reaction scheme (pure condensation reaction). Moreover, the absence of furfuryl alcohol (FOL) in reaction products allows ruling out the FUR hydrogenation.

A variation in C^0_{ANI} did not affect the selectivity of the products, with maximum values of 93.3 % for IME, 6.5 % for FFA, and 0.2 % for TFFA. However, the FFA formation rates (r_{FFA}) sharply increased from 0.672 mmol h^{-1} g^{-1} to 4.8 mmol h^{-1} g^{-1} (Fig. 7b) when C^0_{ANI} changed from 0.125 to 0.5 mol L^{-1}. In addition to the stoichiometric effect of C^0_{ANI} on FFA formation, this increase in reaction rate demonstrates a kinetic effect, which suggests that ANI is a reagent that does not limit the catalytic surface and therefore does not form part of the site balance. Considering that kinetic experiments must be performed under low conversion conditions and far from equilibrium, the tests were henceforth carried out in an ANI deficit.

The change in the initial concentration of FUR decreases its conversion due to the limited amount of ANI available in the medium (0.125 mol/L). ANI conversion did not reflect significant changes with respect to C^0_{FUR} which again confirms their stoichiometric condensation and the absence of FUR direct hydrogenation (Fig. 8a).

The formation of the IME increased with C^0_{FUR}, as evidenced by a remarkable increase in selectivity from 75.1 % (C^0_{FUR} = 0.125 mol L^{-1}) to 93.3 % (C^0_{FUR} = 0.5 mol L^{-1}), this being the most abundant reaction intermediate. However, under the same conditions, the selectivity towards FFA decreased from 25.5 % to 6.5. A plausible hypothesis explaining this reduction in S_{FFA} is supported by a competitiveness between the FUR and the IME for the surface-active sites. This behaviour agrees with the hydrogenation results reported in the control experiments, where acid sites were shown to participate in the adsorption of FUR. The hypothesis is further strengthened by observing that the FFA formation rate values decrease from 2.88 mmol h^{-1} g^{-1} to 0.672 mmol $h^{-1}g^{-1}$ as C^0_{FUR} increases (Fig. 8b), suggesting its involvement as an inhibitor of the hydrogenation step and that it is present in the site balance. The C^0_{FUR} had no effect on further hydrogenation of the furanic ring to form TFFA; in fact, the selectivity was less than 0.5 % for all cases. This agrees with the proposed scheme, where TFFA can only be formed by over-hydrogenation of FFA in a step where FUR is not involved.

Fig. 9a shows that the conversion of FUR depends on H_2 pressure at the conditions studied (0.5–2 bar), reflecting the preference of hydrogen to reduce the C=N bond of the IME rather than the carbonyl group (C=O) of FUR, leading to the formation of FOL.

With respect to selectivity, secondary amine formation was limited at low H_2 pressure (0.5–1 bar) and the predominant product was the IME intermediary with a selectivity of 89 %, mainly attributed to insufficient H_2 in the fluid phase, where it diffuses to adsorb and activate at Pd^0 sites [5]. A further rise in H_2 pressure (from 1 to 2 bar) led to enhanced selectivity for FFA formation, associated with a higher availability of hydrogen to selectively reduce the C=N bond of the IME. Such hydrogenation occurs preferentially as compared to the C=O, and to that of the aromatic ring of the FUR in FFA, as confirmed by the absence of TFFA and FOL in the reaction products. Although H_2 pressure favoured the formation of FFA, its increase does not always guarantee a higher availability of atoms for the reaction, as it is limited by the solubility of hydrogen in the liquid phase and its transfer to the catalyst surface [58].

The kinetic importance of hydrogen is further demonstrated by observing the rates of FFA formation, which are enhanced with increasing pressure (Fig. 9b). At 0.5 bar H_2 the rate was 0.48 mmol h^{-1} g^{-1} and increased to 0.96 mmol h^{-1} g^{-1} with increasing pressure to 2 bar. The results can be attributed to the fact that, at higher hydrogen pressure, the availability of H atoms increased and, with it, the probability of encountering the C=N bond. This accelerated the reduction of

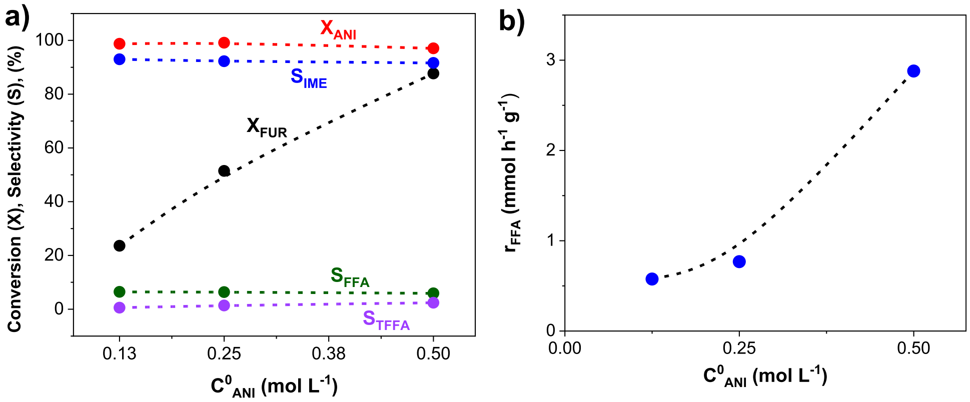

Fig. 7. Effect of the initial ANI concentration on a) conversion and selectivity; b) FFA formation rates. Reactions were carried out for 120 min at T = 50 °C, 0.5 mol L^{-1} of FUR, 1 bar H_2 and substrate/catalyst Pd/ZrO_2-TiO_2 molar ratio = 567.

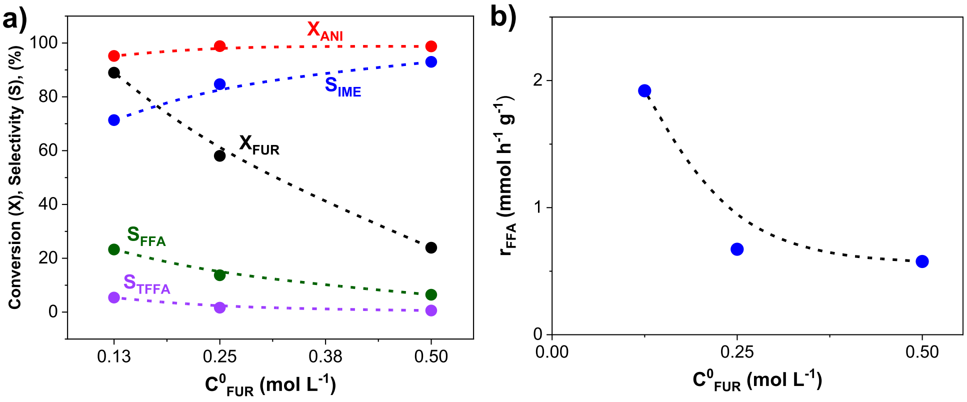

Fig. 8. Effect of the initial FUR concentration on a) conversion and selectivity; b) FFA formation rates. Reactions were carried out for 120 min at T = 50 °C, 0.125 mol L^{-1} of ANI, 1 bar H_2 and substrate/catalyst Pd/ZrO_2-TiO_2 molar ratio = 567.

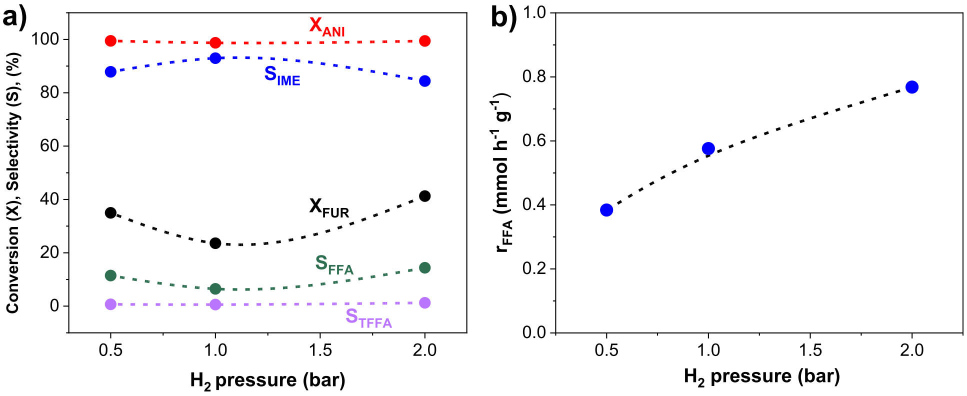

Fig. 9. Effect of the H_2 pressure on a) conversion and selectivity; b) FFA formation rates. Reactions were carried out for 120 min at T = 50 °C, 0.5 mol L^{-1} of FUR, 0.125 mol L^{-1} of ANI and substrate/catalyst Pd/ZrO_2-TiO_2 molar ratio = 567.

the IME to FFA, which is in agreement with what has been previously reported for similar reactions [20,59]. Based on these discussions, it can be suggested that hydrogen is a relevant species in the mechanism of this reaction, forming part of the site balance.

3.6. *Effect of the reaction temperature*

Evaluation of the performance of catalysts at different temperatures provides relevant information on the energy barriers that must be overcome for a reaction to take place, which is a fundamental aspect of the elucidation of reaction mechanisms [23]. Fig. 10 shows the effect of temperature (50–100 °C) on the reductive amination of FUR with ANI.

The FUR conversion reached a plateau at 32.5 % from 75 to 100 °C with respect to temperature (Fig. 10a). This is consistent with our previous calculations on the reaction equilibria and with observations of other authors [15,20,60]. Furthermore, the results allow us to assume that the activation energy in the condensation step is low, which explains the possibility that the reaction occurs rapidly in the homogeneous phase.

However, the increase in the temperature greatly favored the FFA selectivity, reaching up to 26.9 % at 100 °C, similar to that previously reported by Singh et al. [23]. The results are consistent with the Fig. 10b,

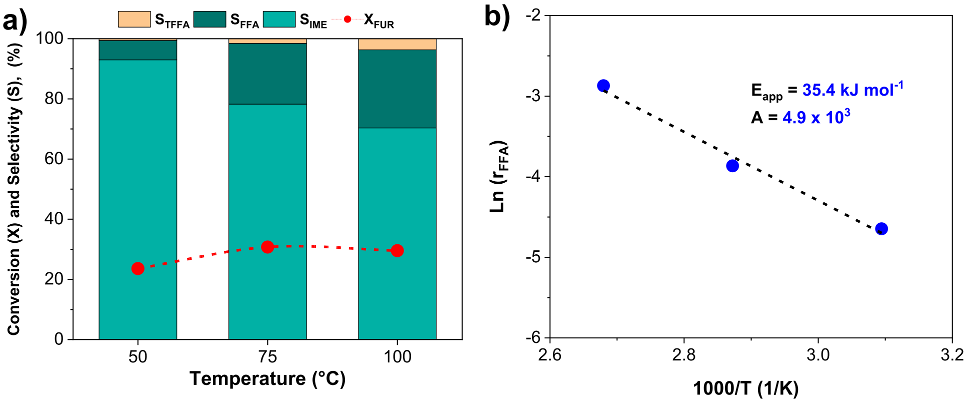

Fig. 10. Effect of the reaction temperature. a) conversion and selectivity; b) Arrhenius plot. Reactions were carried out for 120 min with 0.5 mol L^{-1} of FUR, 0.125 mol L^{-1} of ANI, 1 bar H_2, and substrate/catalyst Pd/ZrO_2-TiO_2 molar ratio = 567.

where the rate of FFA formation increases considerably with increasing temperature. This indicates that the IME hydrogenation step possesses a significantly higher activation energy than its condensation in the first reaction step, according to the proposed scheme. Furthermore, in line with previous discussions in this work, the favourable effect of temperature on FFA selectivity may be associated with lower surface coverage by FUR. Consequently, the competitive adsorption of C═O and C═N groups is reduced, and the availability of sites for IME adsorption increases, as a fundamental step for its subsequent hydrogenation.

In the studied temperature range (50–100 °C), no FOL formation was evident, confirming the selectivity of the catalyst to hydrogenate the C═N group. Furthermore, it suggests that the activation energy for FUR hydrogenation is higher than that required for IME hydrogenation. On the other hand, the limited amount of over-hydrogenated secondary amine (TFFA) in the reaction products suggests that the desorption of FFA is faster than its subsequent hydrogenation. This may be due to the weak interaction between FFA and the active sites, as reported in previous sections.

3.7. Kinetic interpretation of FUR amination

The kinetic effect of the reaction conditions on the amination of FUR was interpreted with a simple kinetic approach using a power law model (Eq. 7).

$$r_i = \left[\left(\frac{1}{w_{cat}}\right)\left(\frac{dC_i}{dt}\right)\right]_{t=0} = \left(\frac{1}{w_{cat}}\right)k \quad \left(C^0_{FUR}\right)^{\alpha}\left(C^0_{PhNH_2}\right)^{\beta}\left(C^0_{H_2}\right)^{\gamma} \tag{7}$$

$$k = A\exp\left(\frac{-E_{app}}{RT}\right) \tag{8}$$

Where E_{app} is the apparent activation energy (kJ mol^{-1}), and R is the universal gas constant (J mol^{-1} K^{-1}). The apparent reaction orders for FUR, ANI, and hydrogen are α, β, and γ, respectively.

Based on previous papers by our group, the hydrogen concentration was calculated using Henry's law. While the apparent reaction orders were estimated by linear regression of the $Ln(r_{FFA})$ vs. $Ln(C_i^0)$ curves (Figure S12a-c). Then, the E_{app} was calculated from the slope of the Arrhenius plot (Fig. 10b), knowing that r_{FFA} is directly proportional to the kinetic constant (k) in Eq. 8.

Thus, the resulting kinetic expression for the amination of FUR on Pd/ZrO_2-TiO_2 is given in Eq. 9.

$$r_{FFA} = \left[\left(\frac{1}{w_{cat}}\right)\left(\frac{dC_i}{dt}\right)\right]_{t=0} = 4.9 \quad x \quad 10^3 \quad \exp\left(\frac{35.4}{RT}\right)\left(C^0_{FUR}\right)^{-0.9}\left(C^0_{ANI}\right)^{1.2}\left(C^0_{H_2}\right)^{0.6} \tag{9}$$

The apparent order for FUR (-0.9) indicates that FFA formation has a stoichiometric dependence on FUR (Eq. 9). The negative order for FUR suggests that this compound should be limiting the catalysts surface enabling a competitive adsorption with the IME intermediate. Consequently, FFA formation depends on FUR, and the reaction rate falls in direct proportion to the initial concentration of FUR. The fractional reaction order for hydrogen (0.6) suggests it is dissociatively adsorbed over Pd^0 sites. Meanwhile, the apparent order of ANI (1.2) agrees with the favourable effect on the reaction rate presented in previous sections and confirms that the stoichiometry of IME formation is FUR/ANI = 1. Moreover, the results allow inferring that the participation of ANI is only in the formation of the Schiff base in the first reaction step according to the proposed scheme.

The apparent activation energy (E_{app}) value was 35.4 kJ mol^{-1} for the FFA formation in the direct amination of FUR on Pd/ZrO_2-TiO_2. Although information on the kinetics of this reaction is limited, the results agree with activation energies for hydrogenating an imine group reported recently in the literature (44 – 58 kJ mol^{-1}) [61,62].

3.8. Catalyst stability test

Catalyst stability was inspected by performing 10 consecutive catalytic cycles [63]. A typical cycle consisted of catalytic reaction (100 ºC, 3 bar H_2, $C^0_{ANI} = C^0_{FUR} = 0.5$ mol L^{-1}, 180 min, substrate/catalyst molar ratio = 212), followed by catalyst separation (filtering-washing with solvent). Moreover, between cycles, the reactant quantities were adjusted to the recovered catalyst mass, thus keeping the same substrate/catalyst molar ratio. An intermediate hot-filtering test was performed on the 5th cycle to inspect if there were some catalytic leaching to the media. Quantitative techniques for measuring reaction performance were the same as stated before in the Materials and Methods section.

The catalysts (fresh and spent-recovered after the 10th cycle) were characterized by thermogravimetric analysis (TGA, 50 mL/min air, 10 ºC/min, up to 600 ºC) to inspect carbon deposition, and by Fourier transformed infrared spectroscopy (FTIR, Nicolet is-20, 32 scans, 4 cm^{-1} resolution) with attenuated total reflection (ATR, Specac Quest, Ge crystal) to analyse the nature of deposited carbonaceous species.

Results from catalytic tests are reported in Fig. 11a. The catalyst showed excellent activity during the 10 reaction cycles, converting more than 90 % of the substrate. The selectivity to the desired product (S_{FFA}) remained relatively stable (68.4 – 64.1 %) for approximately 6 consecutive cycles. However, from cycle 7–10 th the selectivity decreases by about 11 %, which can be attributed to the partial deactivation of the catalyst due to carbon deposition. Carbon deposition can arise from intermediates and products that remain adsorbed over multiple catalytic cycles, thereby facilitating the formation of coke structures that deactivate the catalyst.

The TG curve of the catalysts along with its first derivative are reported in Fig. 11b. Both, fresh and spent catalyst show a slight weight increment (2 – 4 wt%) from ambient temperature to 70 ºC, which is ascribed to the adsorption of gases and to the oxidation of Pd^0 by the O_2 from air. Thereafter, the fresh catalyst was thermally stable, while the spent one underwent a continuous loss of weight loss upon stabilization above 500 ºC. This loss of weight (14 wt%) showed a maximum in the DTG curve centered at 293 ºC, which is ascribed to the decomposition of carbon deposits and its precursors (C_xH_y) on the catalyst surface. These carbonaceous species can be generated from the aromatic structures in secondary amines and imine, which are retained in the catalyst's surface/pores.

Considering the aromatic nature of these coke precursors, the deposited coke could contain high molecular weight polycyclic hydrocarbons, mixtures of aromatic hydrocarbons, or even heteroatoms [64]. In fact, the FTIR analysis (Fig. 11c) of the spent catalyst confirmed the presence of aromatic-like structures by showing a clear signal at 1503 cm^{-1}, associated with the stretching of C═C bonds. In addition, the bands at 1343 cm^{-1} and 1158 cm^{-1} suggest that the molecules deposited on the catalyst also possess nitrogen bonds.

Finally, the hot-filtering test (Fig. 11d) confirmed the catalyst stability and the absence of leaching of active phase to the liquid media. With catalyst, the reaction proceeded rapidly until 60 min, at which point the catalyst was removed to stop the reaction. Between 60 and 90 min, the conversion of FUR and the formation of FFA increased slightly even without catalyst, which can be attributed to the fact that the remaining hydrogen in the fluid phase continued to react. After this time, the reaction stopped, and both conversion and selectivity remained constant.

4. Conclusions

The bifunctional metal-support effect in the reductive amination of FUR with ANI was studied using a Pd/ZrO_2-TiO_2 catalyst which was stable for 6 reaction cycles. A simple kinetic approach was employed, and it was concluded that the formation of the IME intermediary can occur in the homogeneous phase as a primary product by condensation

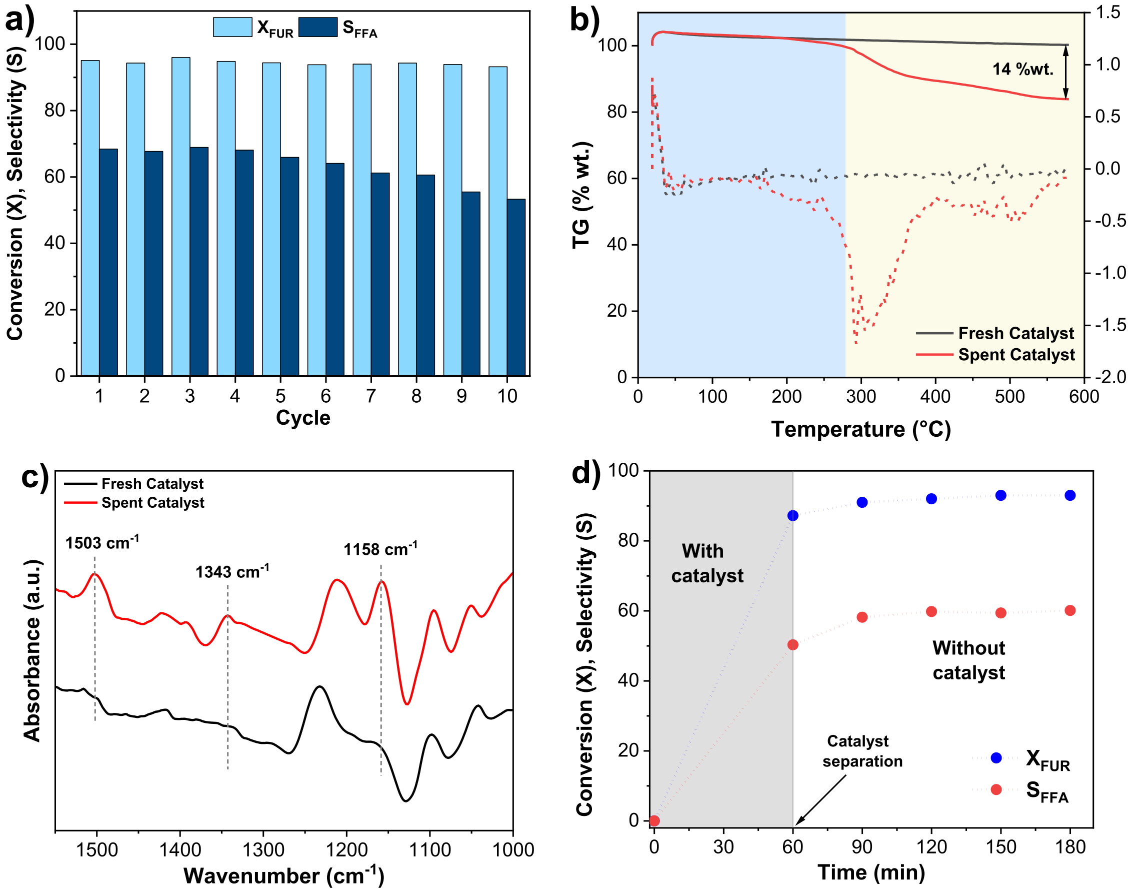

Fig. 11. Results after 10 cycles of reaction: a) catalyst stability test, b) TG and DTG curves, c) FTIR spectra and d) hot-filtering test.

reaction, followed by its catalytic hydrogenation to FFA according to the proposed scheme. The higher reactivity of Pd/ZrO_2-TiO_2 confirmed the involvement of acid sites in the reaction mechanism, particularly in the adsorption of FUR and the IME, where water does not seem to compete for sites. The kinetic interpretation of the results revealed that FUR showed a negative order, indicating its possible participation in the site balance by competing with the IME. While the fractional order of hydrogen revealed the participation of H atoms and their presence in the site balance. This is the first approach to the kinetics of FUR amination with ANI, thus we think these results presents the foundations to other mechanism-oriented studies as well as for preliminary reactor design.

CRediT authorship contribution statement

Daviel Gómez: Writing – review & editing, Visualization, Methodology, Investigation. **Alex A. Fernández-Andrade:** Writing – original draft, Validation, Methodology, Investigation. **John A. Vergara:** Writing – review & editing, Methodology, Formal analysis. **Joan M. Rodríguez-Díaz:** Writing – review & editing, Supervision, Methodology, Conceptualization. **Arteaga Pérez Luis E.:** Writing – review & editing, Supervision, Project administration, Funding acquisition, Conceptualization. **Daniela González-Vera:** Writing – review & editing, Methodology, Investigation. **Cristian H. Campos:** Writing – review & editing, Supervision, Formal analysis, Conceptualization.

Declaration of Competing Interest

The authors declare that they have no known competing financial interests or personal relationships that could have appeared to influence the work reported in this paper.

Acknowledgements

Authors thank ANID-Chile for the financial support granted by the project FONDECYT 1240054. Alex A. Fernández Andrade thanks the financial support of Chilean Scholarship Program ANID BECAS/DOCTORADO NACIONAL 2024/21241495, and to Johnson Matthey for providing $PdCl_2$ through the PGM Award Program.

Appendix A. Supporting information

Supplementary data associated with this article can be found in the online version at doi:10.1016/j.apcata.2025.120455.

Data Availability

Data will be made available on request.

References

[1] P. Zhou, Z. Zhang, L. Jiang, C. Yu, K. Lv, J. Sun, S. Wang, Appl. Catal. B Environ. 210 (2017) 522–532, https://doi.org/10.1016/j.apcatb.2017.04.026.
[2] Ms.K. Saini, S. Kumar, H. Li, S.A. Babu, S. Saravanamurugan, ChemSusChem 15 (2022) e202200107, https://doi.org/10.1002/cssc.202200107.
[3] X. Zhang, S. Xu, Q. Li, G. Zhou, H. Xia, RSC Adv. 11 (2021) 27042–27058, https://doi.org/10.1039/D1RA04633K.
[4] R. Mariscal, P. Maireles-Torres, M. Ojeda, I. Sádaba, M.L. Granados, Energy Environ. Sci. 9 (2016) 1144–1189, https://doi.org/10.1039/C5EE02666K.
[5] D.B. Bagal, R.A. Watile, M.V. Khedkar, K.P. Dhake, B.M. Bhanage, Catal. Sci. Technol. 2 (2012) 354–358, https://doi.org/10.1039/C1CY00392E.
[6] Y. Wu, D. Xu, Y. Xu, X. Tian, M. Ding, Appl. Catal. B Environ. 343 (2024) 123462, https://doi.org/10.1016/j.apcatb.2023.123462.
[7] A. García-Ortiz, J.D. Vidal, M.J. Climent, P. Concepción, A. Corma, S. Iborra, ACS Sustain. Chem. Eng. 7 (2019) 6243–6250, https://doi.org/10.1021/acssuschemeng.8b06631.

[8] M. Hronec, K. Fulajtárová, T. Soták, J. Ind. Eng. Chem. 20 (2014) 650–655, https://doi.org/10.1016/j.jiec.2013.05.029.
[9] M. Hronec, K. Fulajtárová, I. Vávra, T. Soták, E. Dobročka, M. Mičušík, Appl. Catal. B Environ. 181 (2016) 210–219, https://doi.org/10.1016/j.apcatb.2015.07.046.
[10] K.T.V. Yogita, P.M. Rao, N. Kumar, Lingaiah, Sustain. Energy Fuels 6 (2022) 4692–4705, https://doi.org/10.1039/D2SE00408A.
[11] T. Komanoya, T. Kinemura, Y. Kita, K. Kamata, M. Hara, J. Am. Chem. Soc. 139 (2017) 11493–11499, https://doi.org/10.1021/jacs.7b04481.
[12] Y. Yang, L. Zhou, X. Wang, L. Zhang, H. Cheng, F. Zhao, Nano Res. (2022), https://doi.org/10.1007/s12274-022-4923-0.
[13] J. Zhang, J. Yin, X. Duan, C. Zhang, J. Zhang, J. Catal. 420 (2023) 89–98, https://doi.org/10.1016/j.jcat.2023.02.017.
[14] W. Song, Y. Wan, Y. Li, X. Luo, W. Fang, Q. Zheng, P. Ma, J. Zhang, W. Lai, Catal. Sci. Technol. 12 (2022) 7208–7218, https://doi.org/10.1039/D2CY01551J.
[15] C. Dong, H. Wang, H. Du, J. Peng, Y. Cai, S. Guo, J. Zhang, C. Samart, M. Ding, Mol. Catal. 482 (2020) 110755, https://doi.org/10.1016/j.mcat.2019.110755.
[16] C. Dong, Y. Wu, H. Wang, J. Peng, Y. Li, C. Samart, M. Ding, ACS Sustain. Chem. Eng. 9 (2021) 7318–7327, https://doi.org/10.1021/acssuschemeng.1c01456.
[17] T. Komanoya, K. Nakajima, M. Kitano, M. Hara, J. Phys. Chem. C 119 (2015) 26540–26546, https://doi.org/10.1021/acs.jpcc.5b08355.
[18] M.K. Bhunia, D. Chandra, H. Abe, Y. Niwa, M. Hara, ACS Appl. Mater. Interfaces 14 (2022) 4144–4154, https://doi.org/10.1021/acsami.1c21157.
[19] S. Jiang, W. Ramdani, E. Muller, C. Ma, M. Pera-Titus, F. Jerôme, K. De Oliveira Vigiera, ChemSusChem 13 (2020) 1699–1704, https://doi.org/10.1002/cssc.202000003.
[20] N.S. Gould, H. Landfield, B. Dinkelacker, C. Brady, X. Yang, B. Xu, ChemCatChem 12 (2020) 2106–2115, https://doi.org/10.1002/cctc.201901662.
[21] C. Xie, J. Song, M. Hua, Y. Hu, X. Huang, H. Wu, G. Yang, B. Han, ACS Catal. 10 (2020) 7763–7772, https://doi.org/10.1021/acscatal.0c01872.
[22] J. He, L. Chen, S. Liu, K. Song, S. Yang, A. Riisager, Green. Chem. 22 (2020) 6714–6747, https://doi.org/10.1039/D0GC01869D.
[23] G. Singh, J. Kaishyop, G. Singh, Md.J. Gazi, A. Bag, C. Samanta, A. Bordoloi, Mol. Catal. 535 (2023) 112877, https://doi.org/10.1016/j.mcat.2022.112877.
[24] J.J. Martínez, E. Nope, H. Rojas, M.H. Brijaldo, F. Passos, G. Romanelli, J. Mol. Catal. A Chem. 392 (2014) 235–240, https://doi.org/10.1016/j.molcata.2014.05.014.
[25] F. Wang, K. Pan, S. Wei, Y. Ren, H. Zhu, H.-H. Wu, Q. Zhang, Ceram. Int. 47 (2021) 7632–7641, https://doi.org/10.1016/j.ceramint.2020.11.104.
[26] K. Tanabe, T. Sumiyoshi, K. Shibata, T. Kiyoura, J. Kitagawa, Bull. Chem. Soc. Jpn. 47 (1974) 1064–1066.
[27] K.-T. Li, I. Wang, J.-C. Wu, Catal. Surv. Asia 16 (2012) 240–248, https://doi.org/10.1007/s10563-012-9147-y.
[28] R. Rios-Escobedo, E. Ortiz-Santos, J.A. Colín-Luna, J.N. Díaz de León, P. del Angel, J. Escobar, J.A. de los Reyes, Top. Catal. 65 (2022) 1448–1461, https://doi.org/10.1007/s11244-022-01662-x.
[29] F. Lin, X. Jiang, N. Boreriboon, C. Song, Z. Wang, K. Cen, Catal. Today 371 (2021) 150–161, https://doi.org/10.1016/j.cattod.2020.05.049.
[30] A.M. Ruppert, P. Agulhon, J. Grams, M. Wąchała, J. Wojciechowska, D. Świerczyński, T. Cacciaguerra, N. Tanchoux, F. Quignard, Energies 12 (2019) 4706, https://doi.org/10.3390/en12244706.
[31] M. Ortega, D. Gómez, R. Manrique, G. Reyes, J.Tatiana García-Sánchez, V.G. B. Medrano, R. Jiménez, L.E. Arteaga-Pérez, React. Chem. Eng. 8 (2023) 47–63, https://doi.org/10.1039/D2RE00259K.
[32] P.D. Nellist, S.J. Pennycook, Phys. Rev. Lett. 81 (1998) 4156–4159, https://doi.org/10.1103/PhysRevLett.81.4156.
[33] Justin Gorham, NIST X-ray Photoelectron Spectroscopy Database SRD 20 (2012), https://doi.org/10.18434/T4T88K.
[34] M.A. Vannice, Kinetics of Catalytic Reactions, Springer US, Boston, MA, 2005, https://doi.org/10.1007/b136380.
[35] J.T. Scanlon, D.E. Willis, J. Chromatogr. Sci. 23 (1985) 333–340, https://doi.org/10.1093/chromsci/23.8.333.
[36] C.M. Bernt, G. Bottari, J.A. Barrett, S.L. Scott, K. Barta, P.C. Ford, Catal. Sci. Technol. 6 (2016) 2984–2994, https://doi.org/10.1039/C5CY01555C.
[37] M. Ortega, R. Manrique, R. Jiménez, M. Parreño, M.E. Domine, L.E. Arteaga-Pérez, Catalysts 13 (2023) 654, https://doi.org/10.3390/catal13040654.
[38] M. Li, X. Li, G. Jiang, G. He, Ceram. Int. 41 (2015) 5749–5757, https://doi.org/10.1016/j.ceramint.2014.12.161.
[39] J.G. Mahy, S.D. Lambert, R.G. Tilkin, C. Wolfs, D. Poelman, F. Devred, E. M. Gaigneaux, S. Douven, Mater. Today Energy 13 (2019) 312–322, https://doi.org/10.1016/j.mtener.2019.06.010.
[40] F.T.L. Muniz, M. a R. Miranda, C. Morilla dos Santos, J.M. Sasaki, Acta Cryst. A 72 (2016) 385–390, https://doi.org/10.1107/S205327331600365X.
[41] M. Kikugawa, K. Yamazaki, H. Shinjoh, Appl. Catal. A Gen. 547 (2017) 199–204, https://doi.org/10.1016/j.apcata.2017.09.005.
[42] M. Thommes, K. Kaneko, A.V. Neimark, J.P. Olivier, F. Rodriguez-Reinoso, J. Rouquerol, K.S.W. Sing, Pure Appl. Chem. 87 (2015) 1051–1069, https://doi.org/10.1515/pac-2014-1117.
[43] R. Bardestani, G.S. Patience, S. Kaliaguine, Can. J. Chem. Eng. 97 (2019) 2781–2791, https://doi.org/10.1002/cjce.23632.
[44] I.Y. Ahn, W.J. Kim, S.H. Moon, Appl. Catal. A Gen. 308 (2006) 75–81, https://doi.org/10.1016/j.apcata.2006.04.027.
[45] D. Liu, P. Yuan, H. Liu, J. Cai, D. Tan, H. He, J. Zhu, T. Chen, Appl. Clay Sci. 8081 (2013) 407–412, https://doi.org/10.1016/j.clay.2013.07.006.
[46] A.A. Silahua-Pavón, C.G. Espinosa-González, F. Ortiz-Chi, J.G. Pacheco-Sosa, H. Pérez-Vidal, J.C. Arévalo-Pérez, S. Godavarthi, J.G. Torres-Torres, Catal. Commun. 129 (2019) 105723, https://doi.org/10.1016/j.catcom.2019.105723.
[47] J. Bruce, K. Bosnick, E. Kamali Heidari, Sens. Actuators B Chem. 355 (2022) 131316, https://doi.org/10.1016/j.snb.2021.131316.
[48] N. Kruse, S. Chenakin, Appl. Catal. A Gen. 391 (2011) 367–376, https://doi.org/10.1016/j.apcata.2010.05.039.
[49] B. Erdem, R.A. Hunsicker, G.W. Simmons, E.D. Sudol, V.L. Dimonie, M.S. El-Aasser, Langmuir 17 (2001) 2664–2669, https://doi.org/10.1021/la0015213.
[50] C. Sun, L. Liu, L. Qi, H. Li, H. Zhang, C. Li, F. Gao, L. Dong, J. Colloid Interface Sci. 364 (2011) 288–297, https://doi.org/10.1016/j.jcis.2011.07.055.
[51] J. Xiao, Q. Jin, J. Yang, L. Xiong, J. Qiu, J. Jiang, Y. Peng, T. Li, Z. Qiu, W. Yang, Asian J. Org. Chem. 8 (2019) 328–334, https://doi.org/10.1002/ajoc.201800690.
[52] V. Boosa, S. Varimalla, M. Dumpalapally, N. Gutta, V.K. Velisoju, N. Nama, V. Akula, Appl. Catal. B Environ. 292 (2021) 120177, https://doi.org/10.1016/j.apcatb.2021.120177.
[53] M. Lesiak, M. Binczarski, S. Karski, W. Maniukiewicz, J. Rogowski, E. Szubiakiewicz, J. Berlowska, P. Dziugan, I. Witońska, J. Mol. Catal. A Chem. 395 (2014) 337–348, https://doi.org/10.1016/j.molcata.2014.08.041.
[54] C. Wang, Z. Liu, L. Wang, X. Dong, J. Zhang, G. Wang, S. Han, X. Meng, A. Zheng, F.-S. Xiao, ACS Catal. 8 (2018) 474–481, https://doi.org/10.1021/acscatal.7b03443.
[55] R.M. Silverstein, F.X. Webster, D.J. Kiemle, D.L. Bryce. Spectrometric Identification of Organic Compounds, 8th ed., John Wiley & Sons, 2014.
[56] N.A. Bhore, M.T. Klein, K.B. Bischoff, Ind. Eng. Chem. Res. 29 (1990) 313–316.
[57] A. Garcia-Ortiz, J.D. Vidal, S. Iborra, M.J. Climent, J. Cored, D. Ruano, V. Pérez-Dieste, P. Concepción, A. Corma, J. Catal. 389 (2020) 706–713, https://doi.org/10.1016/j.jcat.2020.06.036.
[58] K.L. Deutsch, B.H. Shanks, J. Catal. 285 (2012) 235–241, https://doi.org/10.1016/j.jcat.2011.09.030.
[59] M. Chatterjee, T. Ishizaka, H. Kawanami, Green. Chem. 18 (2016) 487–496, https://doi.org/10.1039/C5GC01352F.
[60] X. Zhuang, J. Liu, S. Zhong, L. Ma, Green. Chem. 24 (2022) 271–284, https://doi.org/10.1039/D1GC03578A.
[61] J.-Y. Yeh, C.-T. Chen, Y.-L. Yang, J.-C. Chen, W.-Y. Yu, Y.-P. Li, K.C.-W. Wu, J. Taiwan Inst. Chem. Eng. 158 (2024) 104884, https://doi.org/10.1016/j.jtice.2023.104884.
[62] H. Gong, L. Wei, Q. Li, J. Zhang, F. Wang, J. Ren, X.-L. Shi, Langmuir 40 (2024) 8950–8960, https://doi.org/10.1021/acs.langmuir.4c00112.
[63] X. Zhuang, J. Liu, S. Zhong, L. Ma, Green. Chem. 24 (2022) 271–284, https://doi.org/10.1039/D1GC03578A.
[64] E.T.C. Vogt, D. Fu, B.M. Weckhuysen, Angew. Chem. Int. Ed. 62 (2023) e202300319, https://doi.org/10.1002/anie.202300319.
